# Freshwater fish condition responses to hydrological disturbance are species‐ and scale dependent

**DOI:** 10.1111/jfb.70033

**Published:** 2025-04-11

**Authors:** Maxwell C. Mallett, Jason D. Thiem, Gavin L. Butler, Luke Carpenter‐Bundhoo, Mark J. Kennard

**Affiliations:** ^1^ Australian Rivers Institute, School of Environment and Science Griffith University Nathan Queensland Australia; ^2^ New South Wales Department of Primary Industries Narrandera Fisheries Centre Narrandera New South Wales Australia; ^3^ New South Wales Department of Primary Industries Grafton Fisheries Centre Trenayr New South Wales Australia

**Keywords:** fish condition, flow regime disturbance, Murray‐Darling Basin, scale dependency

## Abstract

Modification of river flows is a major cause of freshwater fish population declines in many parts of the world. Identifying the precise mechanisms of these declines represents a significant challenge, as a range of stressors can simultaneously impact various components of fish health, fitness and population dynamics. Here we investigate the role of river flows and other biophysical factors on spatio‐temporal variation in freshwater fish body condition in Australia's highly modified Murray‐Darling Basin using three widely distributed native (Murray cod *Maccullochella peelii*, golden perch *Macquaria ambigua* and bony herring *Nematalosa erebi*) and one introduced (common carp *Cyprinus carpio*) species. Our aim was to uncover drivers of spatio‐temporal variation in fish condition at two spatial extents: at the basin scale, utilising a flow regime disturbance index, and at the river‐valley scale, employing individual flow gauge data to assess responses in fish condition to multiple measures of antecedent (365 day) flow. Linear mixed effects modelling revealed that at the basin scale, *M. peelii* and *M. ambigua* were in better condition in rivers with lower flow regime disturbance, and temporal trends in the condition of *N. erebi*, *C. carpio* and *M. peelii* reflected boom and bust dynamics related to wet and dry climate periods. At the river‐valley scale, mean antecedent daily flow magnitude was significantly positively related to the condition of *M. peelii*, *M. ambigua* and *C. carpio*, whereas the number of high‐flow days was negatively related to condition of *N. erebi*. Our study demonstrates that a simple body condition index calculated from routinely collected length–weight data is sensitive to multiple measures of hydrological disturbance in river systems that experience substantial temporal and spatial variability. We emphasise that studies considering multiple spatial scales are important for understanding complex scale‐dependent mechanisms influencing fish condition.

## 1 INTRODUCTION

Human modification of river flow regimes represents a global issue for freshwater biodiversity (Bunn & Arthington, 2002). The combination of water infrastructure to store or divert surface water and associated water extraction or release has substantially altered flow regimes and negatively impacted the ecological integrity of rivers (Arthington et al., 2006). As the life‐history strategies of freshwater organisms, including fish, are intrinsically linked to the natural flow regime of rivers, modification of flow regime components can affect their ability to carry out key life‐history processes (King et al., 2009). Hydrological variability is also a primary driver of freshwater ecosystem productivity, affecting food availability that sustains fishes occupying various trophic niches (Bunn, Thoms, et al., 2006; Junk et al., 1989; Warfe et al., 2011).

Individual fish condition is increasingly being recognised as a crucial factor underpinning a fish's fitness and ability to carry out key life‐history processes (Brosset et al., 2023; Karametsidis et al., 2023). Condition is a commonly used term, generally inferred as a proximate measure of the energetic status of an individual (Milot et al., 2014). There are many ways to assess the condition of an individual (Mallett et al., 2024); however, the most common and straightforward way is the use of morphological condition indices based on mass versus length relationships (Hayes & Shonkwiler, 2001). Morphological condition (hereafter ‘condition’) indices are generally accepted as a surrogate for energetic status, assuming that heavier individuals of a given length are in a better condition due to greater energy reserves (Brosset et al., 2023). Fish responses to a wide variety of ecological processes and stressors have been investigated using condition indices (Mallett et al., 2024; Newbery et al., 2024); however, the ways in which fish condition changes in response to flow variability and anthropogenic flow alteration remain uncertain and have been identified as a critical knowledge gap (Koehn et al., 2019; Koehn et al., 2020).

**FIGURE 1:**
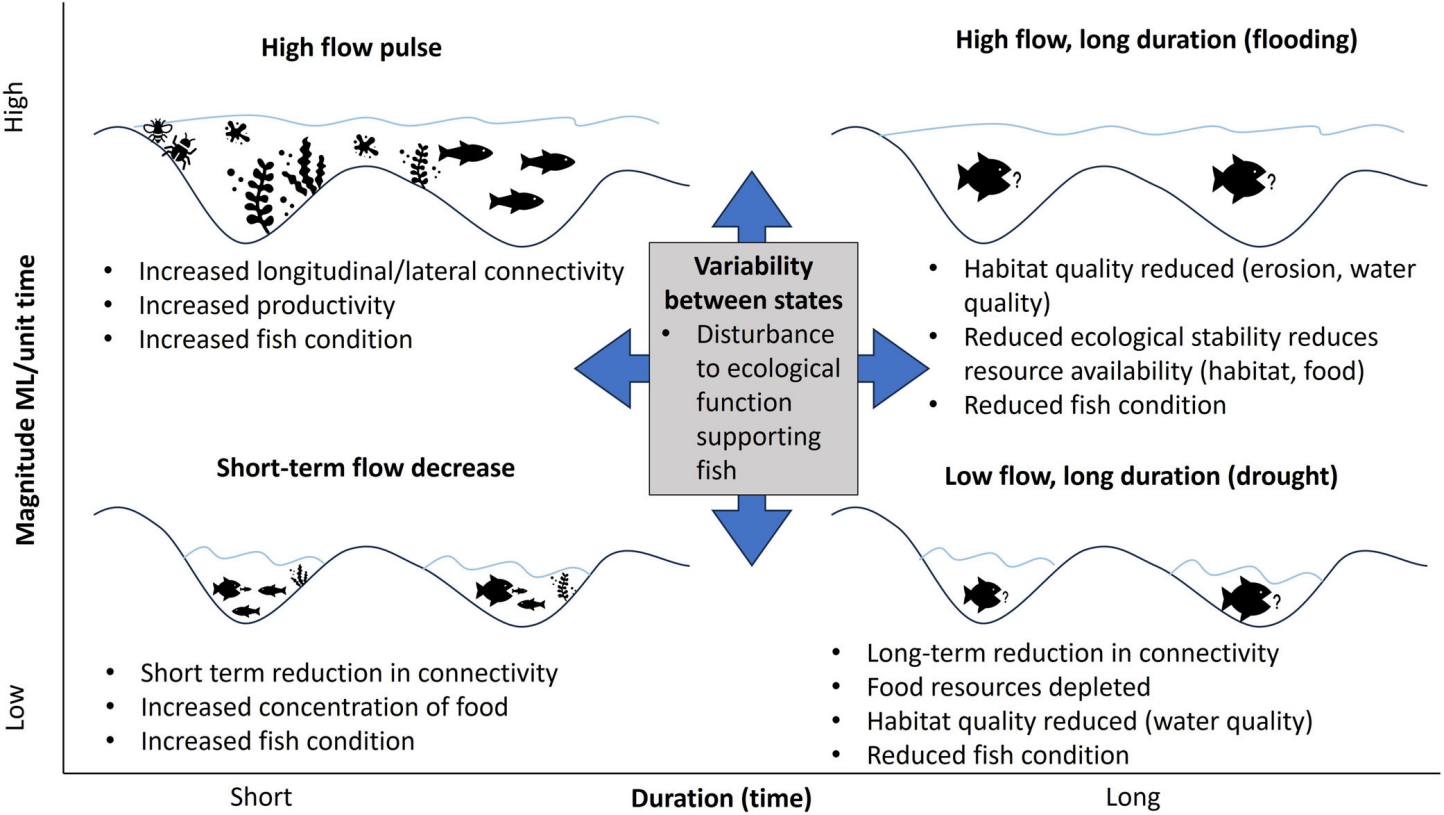
A conceptual diagram of how flow components, including magnitude, duration and variability (encompassing rate of change and frequency), may impact ecological factors and fish condition in river–floodplain systems.

Understanding how fish condition changes in response to river flows may depend on the scale at which these relationships are evaluated (Hewitt et al., 2010; Lowe et al., 2006). At the river‐valley scale, disturbances to flow regime components, such as magnitude, duration, timing, frequency and rate of change, can severely modify rivers, shifting them away from their natural ecological state. These factors collectively influence the availability of local refuges, food resources, opportunities for movement and migration, and affect the condition of resident fish populations (e.g., Arthington et al., 2006; Humphries et al., 1999; Kennard et al., 2007; Magoulick & Kobza, 2003; Schlosser, 1985; Vila‐Gispert & Moreno‐Amich, 2001) (Figure 1). However, one shortcoming of studies attempting to understand the drivers of fish condition at a river‐valley scale is that they cannot determine if these responses are consistent among multiple rivers and if different rivers existing in varying states of broad disturbance display consistent relationships. This knowledge is crucial to fisheries managers overseeing large geographical areas encompassing many river basins (Fausch et al., 2002; Rolls et al., 2023). Furthermore, considering the basin‐scale context that a particular river reach is situated in may be important for determining the degree to which site‐specific environmental variables (e.g., site‐specific flow conditions) will drive changes in fish health. Some studies have suggested that for highly disturbed catchments, site or river‐valley conditions are less important in relation to fish assemblage structure, whereas site‐specific flow conditions in river valleys in less disturbed catchments have a greater impact on fish (Allan et al., 1997; Wang et al., 2006). Outside of the realm of disturbances, rivers that are geographically distinct can also vary in ambient environmental factors such as average water temperature and hydrology, potentially driving differences in fish condition in rivers with large geographical separation (Vila‐Gispert & Moreno‐Amich, 2001).

Australia's Murray‐Darling Basin (MDB) has experienced widespread flow regime modifications due to water resource development and land use change (Stewardson et al., 2020). As a result, freshwater fish populations within the MDB are considered to be in a highly degraded state (Koehn et al., 2014). Key knowledge gaps have been identified pertaining to the links between modified hydrology and fish condition (Koehn et al., 2019). In this study, we quantify the influence of river flow variability, anthropogenic flow alteration and other biophysical factors on fish body condition at two spatial extents within the New South Wales (NSW) MDB. Specifically, this study aims to (1) elucidate the effects of anthropogenic flow regime disturbance on broad‐scale spatial–temporal patterns in fish condition across the NSW MDB and (2) uncover the primary drivers of fish condition at a finer scale in selected river valleys within the NSW MDB. At each spatial extent, we analyse trends in the condition of four freshwater fish species found throughout the NSW MDB: bony herring *Nematalosa erebi* (Günther, 1868), common carp *Cyprinus carpio* L. 1758, golden perch *Macquaria ambigua* Richardson 1845 and Murray cod *Maccullochella peelii* Mitchell 1838. We employ a broad‐scale metric of flow regime disturbance at the basin scale and utilise flow gauge data at the river‐valley scale to uncover fine‐scale responses to multiple flow components. We hypothesised that at a basin scale, fish condition would negatively correlate with flow regime disturbance, whereas at a river‐valley scale, responses in fish condition to flow variability will likely be species‐ (depending on trophic niche and life‐history strategy) and river‐specific (depending on the level of alteration to flow regime components, Figure 1). Findings from this study will aid in determining whether commonly collected morphological metrics (length and weight) are useful for investigating trends in fish condition in response to anthropogenic disturbances and environmental variability.

## 2 METHODS

### 2.1 Study area

**FIGURE 2:**
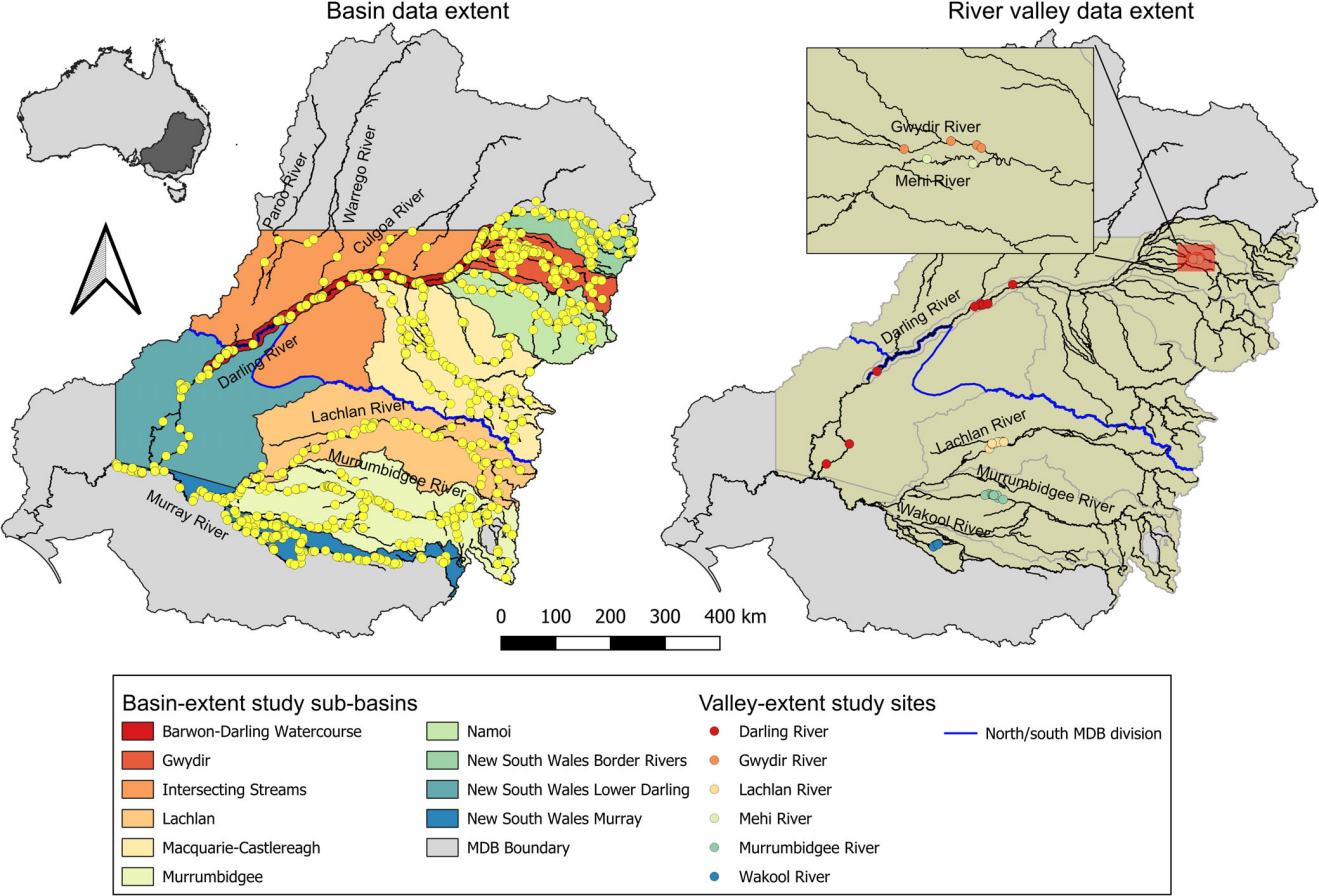
Map depicting the location of fish sampling sites across the New South Wales Murray‐Darling Basin (Australia) spanning two spatial scales (basin extent and river‐valley extent). The blue line represents the boundary between the northern and southern basins. Mehi and Gwydir rivers data were pooled for the river‐valley scale analysis. Descriptions of each river valley are provided in Supporting Information S1.

The NSW portion of the MDB is located in south‐eastern Australia and consists of 10 major sub‐basins (Figure 2), fed by semi‐monsoonal summer rainfall in the northern MDB (NMDB) and winter and spring rainfall in the southern MDB (SMDB) (Koehn et al., 2020; Rolls et al., 2023). A semi‐arid climate dominates much of the MDB to the west, whereas the eastern and southern extents are primarily temperate, and the northern extent is subtropical. From north to south, there is a significant temperature gradient (higher in the north), whereas a rainfall gradient coincides with an elevation gradient from east to west, both being higher in the east (Koehn et al., 2020a). The MDB is often described as a region of ‘booms’ and ‘busts’, largely a result of the highly variable annual rainfall that much of the region receives (Cruz et al., 2020; Koehn et al., 2020; Sternberg et al., 2012). Intense rainfall in the MDB can trigger extensive flooding that promotes significant increases in productivity. This rainfall is most often interspersed among extended periods of severe drought that can persist for multiple years, causing significant declines in biodiversity across all river scales. Within the time frame of the current study, the MDB experienced both severe drought between the years 2017 and 2019 and flooding in multiple rivers in 2016, 2020 and 2021 (Figure S1) (Bureau of Meteorology, 2020).

### 2.2 Study species

To assess the impacts of hydrological variability and anthropogenic flow alteration on fish health in the MDB, we selected four species that display a variety of ecological traits and life‐history strategies: three native species – *N. erebi*, *M. ambigua* and *M. peelii*, and *C. carpio*, an introduced species from Eurasia (Tonkin et al., 2021). The life‐history strategies of the selected species represent two of three commonly recognised guilds: equilibrium (*M. peelii*) and periodic (*M. ambigua*, *C. carpio* and *N. erebi*) (Butler et al., 2022; Kopf et al., 2014; Winemiller & Rose, 1992). This trait‐based approach is considered best practice in riverine fish monitoring programmes and allows inferences regarding species of a particular guild to be transferred to other species in that guild that share similar traits (Rose et al., 2015).

### 2.3 Fish condition index and demographic database

Field‐collected demographic data, including length (using a standard field measuring board), weight (using a set of electronic balance scales) and catch location (latitude and longitude obtained via GPS), were provided by NSW Department of Primary Industries (Fisheries) and also retrieved online from the Commonwealth Environmental Water Holder (CEWH) Flow‐MER Program online database (CEWH, 2023). The data were collected across numerous programmes within the NSW section of the MDB between 2015 and 2021. For the basin‐scale analysis component of our study, the dataset covered a large spatial area, with numerous sites in each sub‐basin spanning headwater to lowland reaches in the NSW section of the MDB. For the river‐valley analysis, the dataset included five river systems for which there was a consistent sampling regime at repeated sites across multiple years and represented the geographic extents of the NSW MDB. This included data from the Lachlan, Murrumbidgee, Edward‐Wakool, Darling and Gwydir (including the Mehi River) river systems.

Fish condition was calculated using a standardised residual condition index (hereafter referred to as condition) as described by Pope and Kruse (2007). For each species, a linear regression of the log‐transformed length and weight values was calculated, with this regression including data for each species gathered from sites spanning the entire NSW MDB (Figure 2), therefore likely being reflective of complete population ranges. For each fish, the difference between the actual weight of the fish and the expected weight from the linear regression was calculated. These values were then standardised and mean‐centred for each species. Because this index is a residual, it is not correlated with body size and therefore is not subject to assumptions in relation to isometric growth (Kaufman et al., 2007; Pope & Kruse, 2007).

Additional covariates potentially explaining variation in body condition were derived from the demographic database. These included catch per unit of effort (CPUE) of all species collected using electrofishing on each sampling occasion. We used CPUE to account for potential density‐dependent effects on fish condition, such as resource limitation due to intraspecific competition (Stoffels et al., 2018). Potential spawning status was considered to account for seasonal differences in condition that can confound interpretation (Blackwell et al., 2000) and was generated for each species based on their known spawning period for a given region and month of capture (Table 1; Humphries, 2005; Koehn et al., 2020; Lintermans, 2023). Length quartiles (Q1–Q4) were generated for each species, at both spatial extents, to account for potential differences in condition in relation to fish size. We avoided the use of other size categories such as length at age, as this is known to be highly variable among species (Anderson et al., 1992; Wright et al., 2020). Total length was used for *M. ambigua* and *M. peelii*, and fork length was used for *N. erebi* and *C. carpio*.

**TABLE 1:** Details of the basin and river‐valley scale dataset used for each species, including number of individuals, number of sites, number of basins, length quartiles, weight range (mean value in brackets) and spawning period.

	*Nematalosa erebi*	*Cyprinus carpio*	*Macquaria ambigua*	*Maccullochella peelii*
Basin scale				
Number of individuals	11,946	14,594	2784	5400
Number of sites	327	634	348	317
Number of rivers	67	152	70	56
Number of basins	10	10	10	9
Length quartile 1 (Q1) range (mm)	36–104	51–174	68–313	50–261
Length quartile 2 (Q2) range (mm)	105–129	175–414	314–395	262–396
Length quartile 3 (Q3) range (mm)	130–191	415–497	396–440	397–505
Length quartile 4 (Q4) range (mm)	192–478	498–820	441–588	506–1220
Weight range (mean) (g)	1.1–1704 (123)	2.9–11,340 (1435)	4.5–4449 (939)	1.5–27,450 (1389)
Valley‐scale				
Number of individuals	15,599	5250	1413	2162
Length quartile 1 (Q1) range (mm)	36–73	44–129	60–371	50–220
Length quartile 2 (Q2) range (mm)	74–92	130–306	372–418	221–341
Length quartile 3 (Q3) range (mm)	93–120	307–468	419–452	342–506
Length quartile 4 (Q4) range (mm)	121–432	498–820	453–571	507–1140
Weight range (mean) (g)	1.1–1279 (43)	1.8–11,340 (1029)	1.5–3642 (1157)	1.5–23,690 (1505)
Spawning period (month number – both extents)	Whole basin: 10, 11, 12	Whole basin: 1,2, 9, 10, 11, 12	Northern basin: 1, 2, 3, 4, 10, 11, 12 Southern basin: 1, 2, 10, 11, 12	Northern basin: 9, 10 Southern basin: 10, 11, 12

*Note*: Designated spawning periods are split across northern and southern basins for *Maccullochella peelii* and *Macquaria ambigua* and are taken from Humphries (2005), Koehn et al. (2020) and Lintermans (2023).

### 2.4 Basin‐scale environmental data

Geospatial data were obtained from the Australian Hydrological Geospatial Fabric (Geofabric) (Bureau of Meteorology, 2010). This dataset includes metrics such as surface water connectivity, catchment and river disturbances, primary productivity and climate variables (Stein et al., 2002). These attributes are provided for river network segments, which are generated based on specific surface features (Stein et al., 2014). For our basin‐scale analysis, two metrics from the Geofabric database were used; the flow regime disturbance index (FRDI) and catchment average annual mean temperature (average temperature), which is the mean annual temperature (°C) of the catchment upstream from the relevant stream segment. The demographic database was attributed to Geofabric river network segments based on latitude and longitude of catch using QGIS (QGIS Development Team, 2024). Then, based on these river segments, FRDI and average temperature were incorporated into the fish demographic database.

**FIGURE 3:**
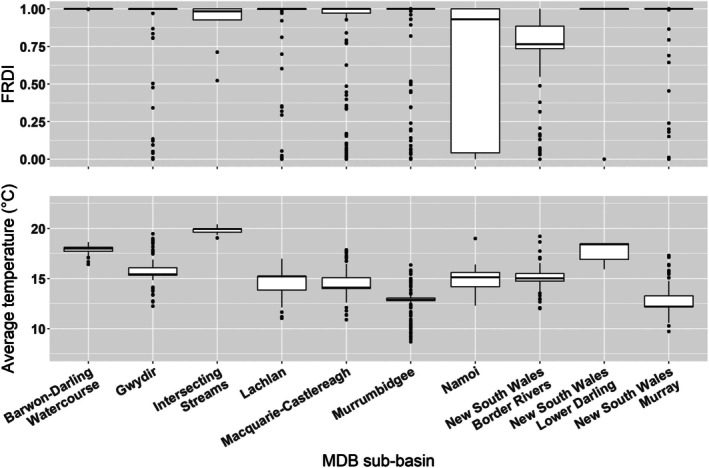
Boxplots of the flow regime disturbance index (FRDI) (top) and average temperature (°C) (bottom) for each Murray‐Darling Basin (MDB) sub‐basin included in our basin‐scale analysis. FRDI values closer to 1 represent higher levels of disturbance.

FRDI was chosen as a measure of in‐stream alterations to the flow regime due to the presence of water infrastructure. Values closer to 1 indicate a more severe alteration (Raupach et al., 2001; Stein et al., 2002). FRDI utilises three separate indices of in‐channel disturbance: the impoundment factor, the flow diversion factor and the levee bank factor (Stein et al., 2002). The impoundment factor considers the presence of structural barriers including dams and weirs. These structures have direct impacts on biota by restricting movement and fragmenting populations, while also limiting the transport of particulate matter (Jager et al., 2001; Kelly, 2001). The flow diversion factor is a measure of alterations to the flow regime due to diversions or additions of in‐stream discharge resulting from the presence of infrastructure related to hydroelectric power generation, irrigation or drainage (Stein et al., 2002). As a consequence of this infrastructure, flow diversions can have potentially severe ecological implications such as altered thermal characteristics, changes in nutrient concentrations or the introduction of exotic species (Davies et al., 2000; Gibbins et al., 2000). Levees banks are artificial embankments, which effectively increase channel depth and prevent overland flow outside of a river channel. The levee bank factor as part of the FRDI used a weighted distance function from the river course with a threshold of 500 m (Stein et al., 2002). Levee banks reduce floodplain connectivity and ultimately resource availability, potentially impacting the ability of fish to maintain good condition (Cruz et al., 2020; Knox et al., 2022). Boxplots of FRDI and average temperature for each NSW MDB sub‐basin are provided in Figure 3. FRDI ranged considerably between sub‐basins but was generally high, with the Barwon‐Darling Watercourse sub‐basin having the highest mean FRDI and the Namoi sub‐basin having the lowest mean FRDI.

### 2.5 River‐valley scale environmental data

**FIGURE 4:**
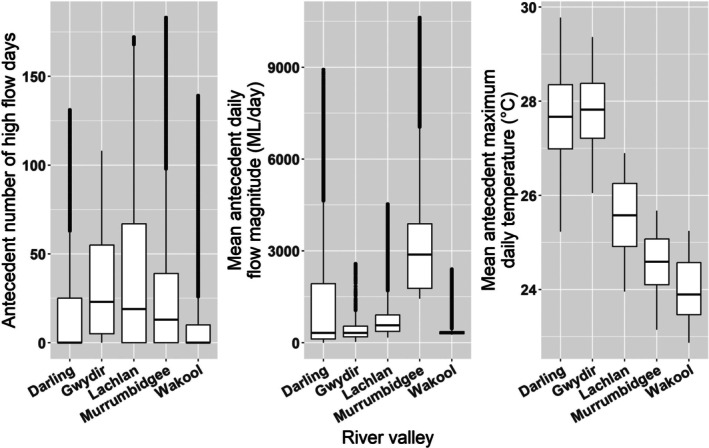
Boxplots showing (from left to right) the antecedent number of high‐flow days (above the 90th percentile of daily flow magnitude), mean antecedent daily flow magnitude (ML/day) and mean antecedent maximum daily temperature (°C) for the river valleys included in our river‐valley scale analysis. Antecedent mean values are calculated over a 365‐day period for the years 2015–2021.

The river‐valley scale dataset included variables from the basin‐scale dataset (condition, spawning status and FRDI), with the exception of the long‐term average temperature and basin‐scale‐specific length quartiles. Separate length quartiles were calculated for each species for our river‐valley scale analysis, and mean antecedent maximum daily temperature (°C) was calculated for the 365‐day antecedent period from the day of fish capture, using daily maximum temperature data from the closest available weather station. We calculated flow variables that reflected the magnitude and duration of flow. All flow metrics were calculated for a 365‐day antecedent period from the date each fish was captured (Balcombe et al., 2012; Tonkin et al., 2011; Tonkin et al., 2017). We calculated the number of days with flow above the 90th percentile (antecedent number of high‐flow days) and the mean antecedent daily flow magnitude. For all rivers, except the Darling River, sites were within 100 km to the gauge location. For the Darling River, sites were distributed across a larger area, meaning data from multiple gauges were needed to calculate flow metrics at each collection of sites. Flow metrics were calculated using data sourced from the WaterNSW website, with missing data for periods <8 days interpolated using linear interpolation. Flow and temperature gauge details are provided in Table S1. Our calculated flow and temperature metrics differed between river valleys (Figure 4). The Lachlan River had the highest mean antecedent number of high‐flow days, whereas the Darling River had the lowest. The Murrumbidgee River exhibited the highest mean antecedent daily flow magnitude, whereas the Gwydir River had the lowest. Mean antecedent maximum daily temperatures closely followed a latitudinal gradient and were highest in more northern river valleys (e.g., Darling and Gwydir) and lowest in more southern river valleys (e.g., Lachlan, Murrumbidgee and Wakool).

### 2.6 Data analysis

We developed linear mixed effects models to investigate the effects of factors relating to environmental variability and disturbance on fish condition in the *lme4* package in R (R Development Core Team; www.r-project.org). Individual models were developed for each species of interest at both basin and river‐valley scales. For our basin‐scale analyses, predictor variables included flow regime disturbance (FRDI), biological factors (spawning period and length quartiles), geographical factors (latitude), climatic factors (average temperature) and year. The basin‐scale dataset consisted of multiple sub‐basins, so we included sub‐basin as a random intercept. For our river‐valley scale analyses, predictor variables included mean antecedent daily flow magnitude, antecedent number of high‐flow days, mean antecedent maximum daily temperature, total fish CPUE, year, spawning status, FRDI, river valley and length quartile. Site was included as a random intercept. Continuous predictor variables for basin and river‐scale analyses were centred and scaled for model convergence. For each model, we did not include interaction terms due to model convergence issues and to avoid model overfitting.

All possible models were investigated, and the best model for each species and scale was selected using the ‘*MuMin*’ package (Barton & Barton, 2015). Marginal and conditional R^2^ values were calculated using the ‘*Performance*’ package (Nakagawa & Schielzeth, 2013). Potential correlation between all predictor variables was checked before their inclusion in the models, and where two or more metrics were highly correlated (at r > 0.75), only one variable was included in the model [similar to previous studies such as Balcombe et al. (2014)]. Models were checked for normality and homoscedasticity as per Zuur et al. (2010). As there were no confirmed recaptures in this dataset, we assume that each observation was temporally independent of one another. Spatial autocorrelation was not found in any of the models. This was first checked using bubble plots of model residuals, then by calculating Moran's I using the ‘*ape*’ package.

## 3 RESULTS

### 3.1 Trends in fish condition at a basin scale

**FIGURE 5:**
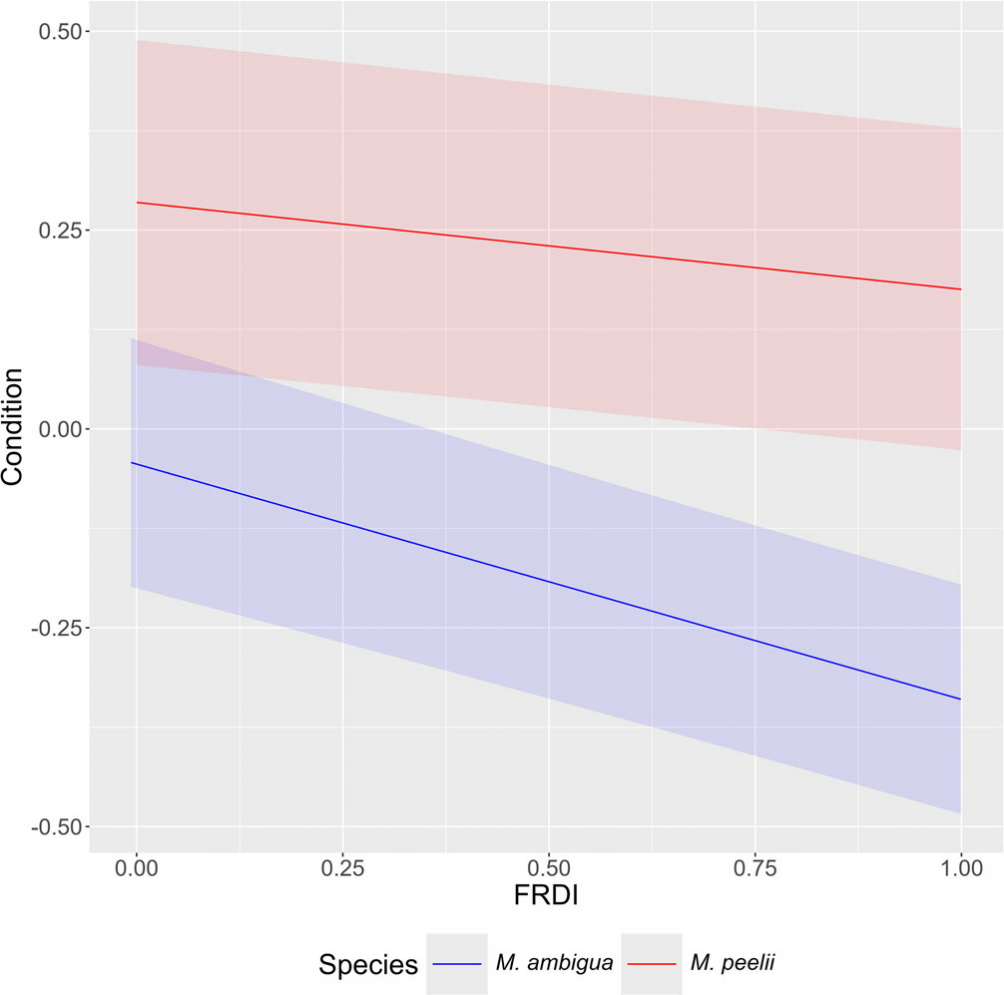
A partial residual plot of the predicted condition [± standard error (SE)] of *Macquaria ambigua* and *Maccullochella peelii* in response to the flow regime disturbance index (FRDI) from basin‐scale models. Probabilities are generated from each species' linear mixed effects model, with all other covariates held at their mean values. FRDI has been unscaled for plotting.

**FIGURE 6:**
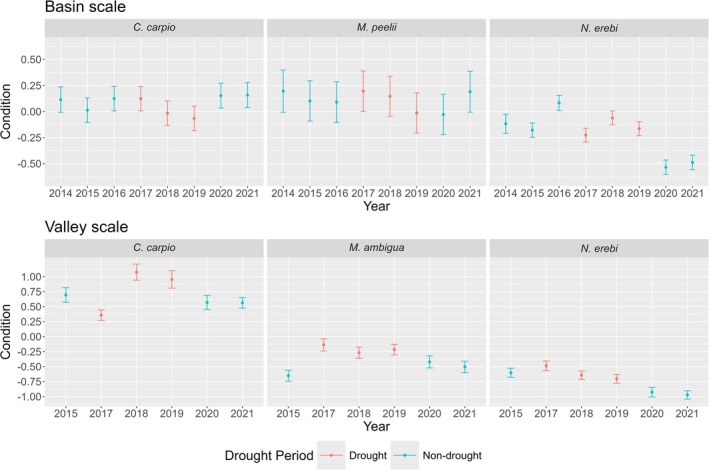
Predicted condition of fish species from basin‐scale (top) and river‐valley scale (bottom) models for years 2014–2021. Years are colour coded to show drought (red) and non‐drought (blue) periods (Bureau of Meteorology, 2020). Confidence bars represent standard error. Probabilities are generated from each species' linear mixed effects model, with all other covariates held at their mean values.

**FIGURE 7:**
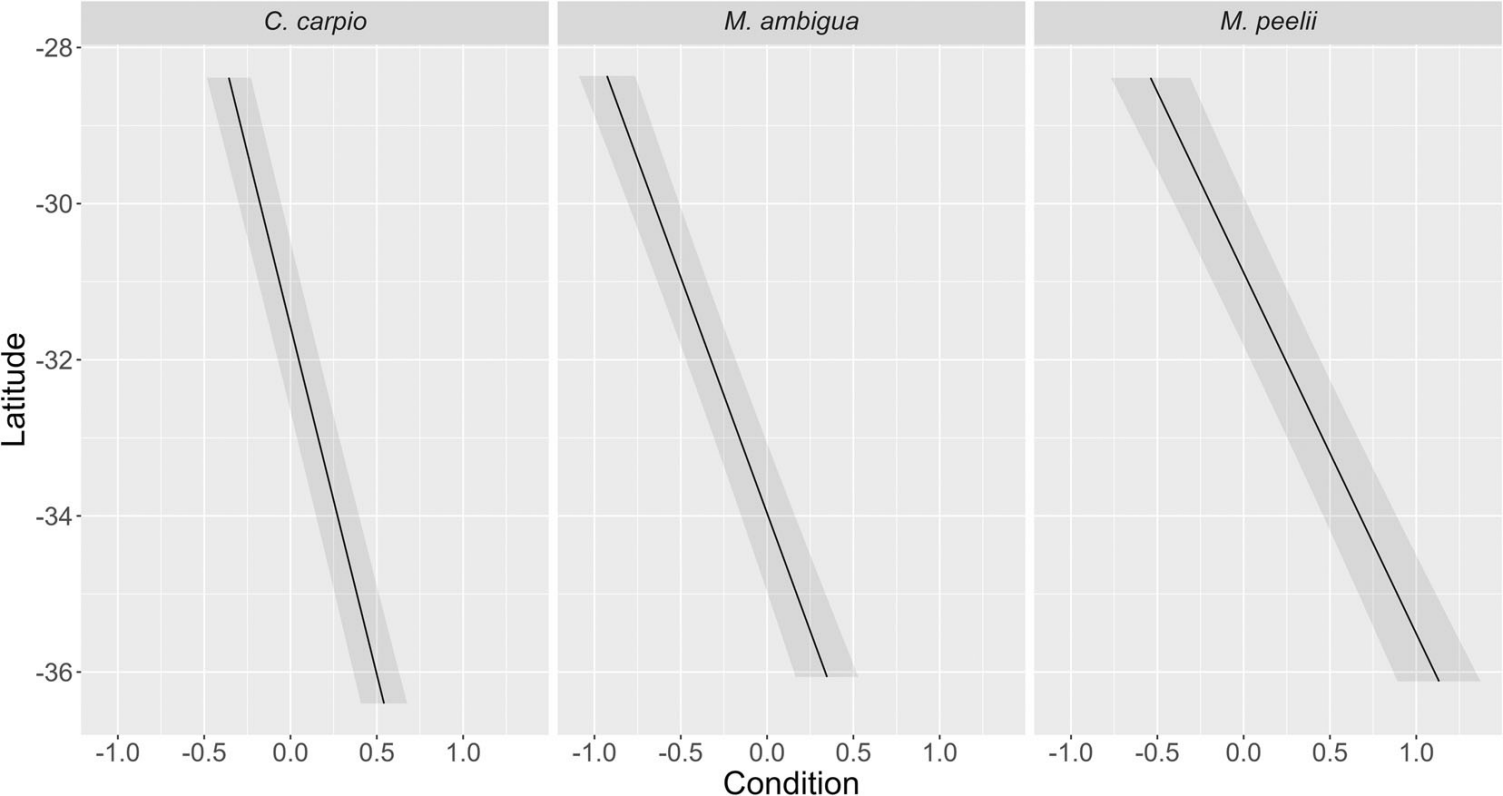
A partial residual plot of the predicted condition [± standard error (SE)] of *Cyprinus carpio, Macquaria ambigua* and *Maccullochella peelii* across a latitudinal gradient for our basin‐scale models. Probabilities are generated from each species' linear mixed effects model, with all other covariates held at their mean values. Latitude has been unscaled for plotting.

**FIGURE 8:**
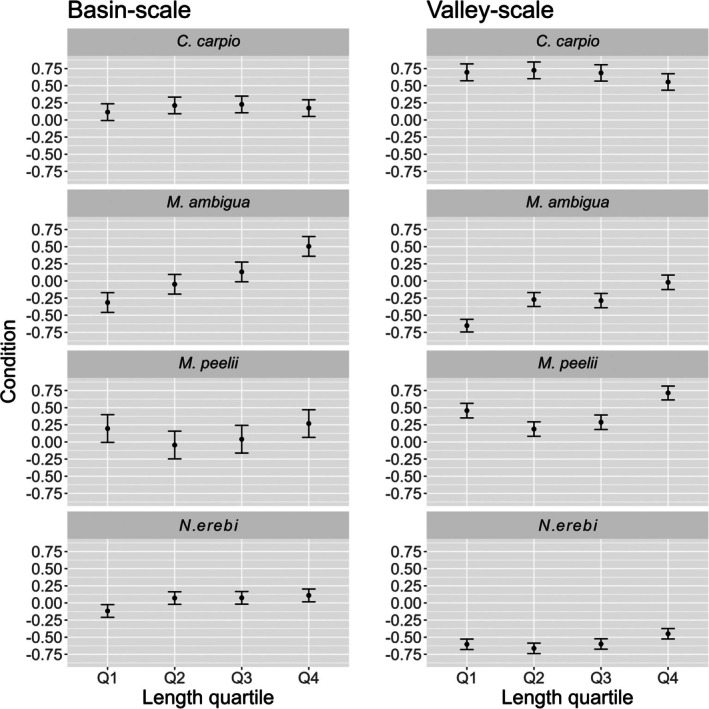
Plots showing the predicted condition based on our basin‐scale (left) and river‐valley scale (right) models across length quartiles (Q1–Q4). Confidence bars represent the standard error. Probabilities are generated from each species' linear mixed effects model, with all other covariates held at their mean values.

Basin‐scale linear mixed effects results for each species are provided in Table 2. The condition of *M. peelii* and *M. ambigua* had a significant relationship with flow regime disturbance at a basin scale (Figure 5). There was strong interannual variation in the condition of *M. peelii*, *C. carpio* and *N. erebi*. *M. peelii* condition was lowest in 2019 and 2020, whereas *C. carpio* condition was lowest in 2018 and 2019, both coinciding with severe drought conditions. *N. erebi* condition was highest in 2016 and lowest in 2020 and 2021, immediately following the drought (Figure 6). There was a latitudinal gradient in fish condition at a basin scale, with *M. peelii*, *M. ambigua* and *C. carpio* in better condition at more southern (more negative) latitudes (Figure 7). *N. erebi* condition did not vary latitudinally (Table 2). *M. peelii*, *M. ambigua* and *N. erebi* belonging to Q4 were in significantly better condition compared to the other size classes, whereas *C. carpio* within Q3 were in significantly better condition than all other size classes (Figure 8; see Table S2 for pair‐wise comparisons of length quartiles). The effect of spawning status on fish condition varied between species, with *C. carpio* and *N. erebi* captured during their respective spawning periods found to be in significantly better condition compared to those captured outside of these periods. Conversely, *M. ambigua* were in significantly worse condition during their spawning period. *C. carpio* and *N. erebi* were found to be in significantly poorer condition at warmer average catchment temperatures.

**TABLE 2:** Linear mixed effect model results for our basin‐scale analysis of drivers of condition in *Nematalosa erebi*, *Cyprinus carpio*, *Macquaria ambigua* and *Maccullochella peelii*.

Basin scale	*N. erebi*		*C. carpio*		*M. ambigua*		*M. peelii*	
	Estimate	*p*	Estimate	*p*	Estimate	*p*	Estimate	*p*
Average temperature	−0.111	**<0.005**	−0.080	**<0.005**				
FRDI					−0.070	**<0.005**	−0.035	**<0.005**
Q2	0.189	**<0.005**	0.097	**<0.005**	0.267	**<0.005**	−0.241	**<0.005**
Q3	0.192	**<0.005**	0.112	**<0.005**	0.447	**<0.005**	−0.156	**<0.005**
Q4	0.227	**<0.005**	0.059	**<0.005**	0.819	**<0.005**	0.073	**<0.005**
Latitude			−0.266	**<0.005**	−0.400	**<0.005**	−0.558	**<0.005**
Spawning period (1)	0.140	**<0.005**	0.086	**<0.005**	−0.224	**<0.005**		
2015	−0.061	>0.05	−0.101	**<0.05**			−0.093	>0.05
2016	0.200	**<0.05**	0.010	>0.05			−0.105	>0.05
2017	−0.109	>0.05	0.009	>0.05			0.000	>0.05
2018	0.056	>0.05	−0.130	**<0.005**			−0.049	>0.05
2019	−0.047	>0.05	−0.181	**<0.005**			−0.208	**<0.005**
2020	−0.418	**<0.005**	0.039	>0.05			−0.223	**<0.005**
2021	−0.371	**<0.005**	0.044	>0.05			−0.005	>0.05
R^2^_c_/R^2^_m_	0.124/0.062		0.318/0.151		0.544/0.366		0.639/0.309	

*Note*: Spawning period (1) represents fish captured within their spawning period. Conditional (R^2^_c_) and marginal (R^2^_m_) R^2^ values are provided in the bottom row. The level of significance is highlighted by the use of “0.05” or “<0.005” (in bold).

Abbreviation: FRDI, flow regime disturbance index.

### 3.2 Trends in fish condition at a river‐valley scale

**FIGURE 9:**
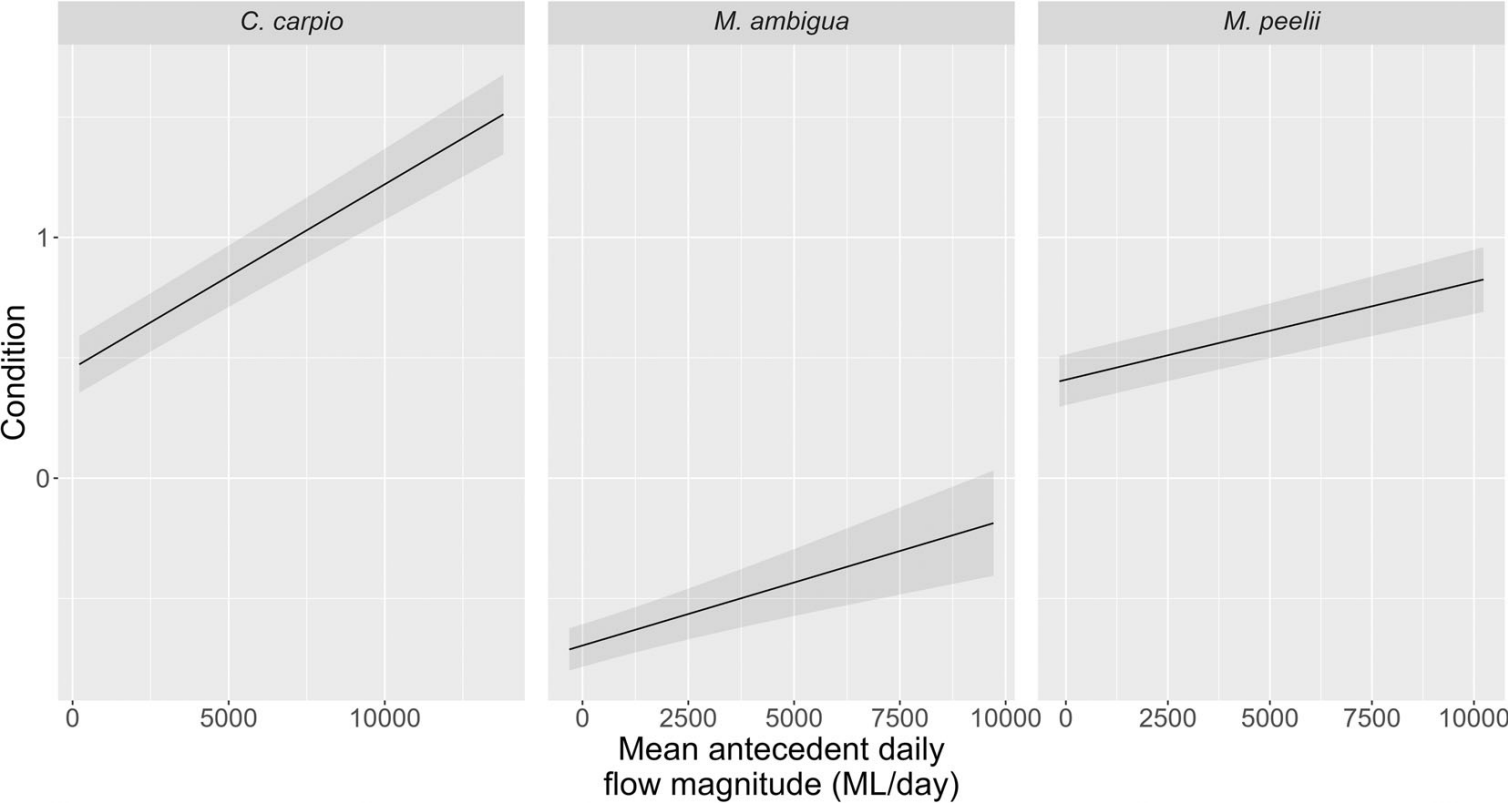
A partial residual plot showing predicted condition [± standard error (SE)] of *Cyprinus carpio, Macquaria ambigua* and *Maccullochella peelii* in response to mean antecedent (365 days) daily flow magnitude (ML/day). Probabilities are generated from each species' linear mixed effects model, with all other covariates held at their mean values. Mean antecedent flow has been unscaled for plotting.

River‐valley scale linear mixed effects results for each species are provided in Table 3. At the river‐valley scale, the condition of *M. peelii*, *M. ambigua* and *C. carpio* was positively related to mean antecedent flow magnitude (Figure 9). The number of high‐flow days in the antecedent period prior to fish capture had a significant negative effect on the condition of *N. erebi*. We found evidence of length‐related differences in condition for each species: *M. peelii*, *M. ambigua* and *N. erebi* within Q4 were in significantly better condition than other size classes, whereas *C. carpio* within Q4 were in significantly worse condition than other size classes (Figure 8; see Table S2 for pair‐wise comparisons of length quartiles).

**TABLE 3:** Linear mixed effect model results for our valley‐scale analysis of drivers of condition in *Nematalosa erebi*, *Cyprinus carpio, Macquaria ambigua* and *Maccullochella peelii*.

Valley scale	*N. erebi*		*C. carpio*		*M. ambigua*		*M. peelii*	
	Estimate	*p*	Estimate	*p*	Estimate	*p*	Estimate	*p*
Q2	−0.059	**<0.005**	0.029	>0.05	0.381	**<0.005**	−0.269	**<0.005**
Q3	0.004	>0.05	−0.009	>0.05	0.366	**<0.005**	−0.170	**<0.005**
Q4	0.153	**<0.005**	−0.141	**<0.005**	0.632	**<0.005**	0.258	**<0.005**
Mean antecedent maximum daily temperature			−0.503	**<0.005**				
Mean antecedent daily flow magnitude			0.198	**<0.005**	0.100	**<0.05**	0.081	**<0.005**
Antecedent # high‐flow days	−0.160	**<0.005**						
Spawning period (1)	0.249	**<0.005**			0.163	**<0.05**		
CPUE	−0.092	**<0.005**	0.066	**<0.005**	0.115	**<0.005**	0.256	**<0.005**
River valley: Gwydir	0.632	**<0.005**	0.488	**<0.005**	−0.356	**<0.05**	−0.366	**<0.005**
River valley: Lachlan	0.803	**<0.005**	−0.615	**<0.005**	0.126	>0.05	−0.104	>0.05
River valley: Murrumbidgee	0.677	**<0.005**	−1.222	**<0.005**	−0.521	**<0.005**	−0.740	**<0.005**
River valley: Wakool	−0.060	>0.05	−0.951	**<0.005**	0.507	**<0.005**	−0.440	**<0.005**
Year: 2017	0.117	>0.05	−0.337	**<0.005**	0.514	**<0.005**		
Year: 2018	−0.040	>0.05	0.381	**<0.005**	0.384	**<0.005**		
Year: 2019	−0.101	**<0.005**	0.260	**<0.005**	0.435	**<0.005**		
Year: 2020	−0.323	**<0.005**	−0.124	**<0.05**	0.231	**<0.005**		
Year: 2021	−0.369	**<0.005**	−0.131	**<0.05**	0.147	**<0.05**		
R^2^_c_/R^2^_m_	0.187/0.145		0.138/0.119		0.426/0.377		0.332/0.306	

*Note*: Spawning period (1) represents fish captured within their spawning period. Conditional (R^2^_c_) and marginal (R^2^_m_) R^2^ values are provided in the bottom row. The level of significance is highlighted by the use of “0.05” or “<0.005” (in bold).

Abbreviation: CPUE, catch per unit of effort.

**FIGURE 10:**
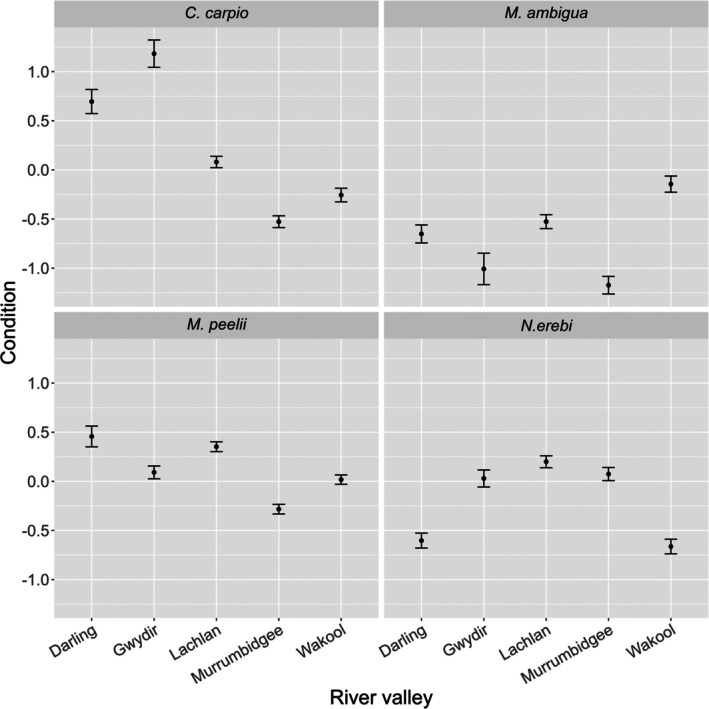
Predicted condition of our study species between river valleys as part of our river‐valley scale analysis. Confidence bands represent the standard error. Probabilities are generated from each species' linear mixed effects model, with all other covariates held at their mean values.

The condition of each species varied considerably between river valleys (Figure 10). The condition of *C. carpio*, *M. ambigua* and *M. peelii* was lowest in the Murrumbidgee River compared to all other river valleys, whereas their condition was highest in the Gwydir, Wakool and Darling rivers, respectively. *N. erebi* condition was lowest in the Wakool River and highest in the Lachlan River compared to all other river valleys. We found that the predicted condition of *N. erebi* followed a similar temporal pattern to our basin‐scale results, being lowest in 2021 and highest in 2017 (Figure 6). The predicted condition of *C. carpio* varied considerably between years, with condition being lowest in 2017 and highest in 2018. *M. ambigua* condition was lowest in 2015 and highest in 2017.

Other variables driving fish condition at the river‐valley scale included CPUE, which had a significant positive relationship to the condition of *M. peelii*, *M. ambigua* and *C. carpio*, and a significant negative relationship to *N. erebi*. Mean maximum daily temperature had a significant negative impact on the condition of *C. carpio*. *M. ambigua* and *C. carpio* captured during their spawning periods were in significantly better condition than those captured outside their known spawning periods.

## 4 DISCUSSION

### 4.1 Flow regime disturbances and fish condition at a basin scale

At a basin scale, we quantified the collective impact of water infrastructure, including dams, levees and water extraction, using a disturbance index, and found that the individual condition of *M. peelii* and *M. ambigua* significantly decreased as flow regime disturbance increased. The mechanisms by which these disturbances affect fish condition are complex but are ultimately related to how they influence the acquisition and expenditure of energy reserves via altered productivity, resource availability and movement requirements (Baumgartner, 2007; Bice et al., 2016; Richardson et al., 2002). Increased energy expenditure as a result of flow regime disturbances has been shown previously to impact energetic status, resulting in decreased growth. In a study investigating the impacts of flow alteration on the growth of *M. peelii* in the SMDB using bioenergetic models, Whiterod et al. (2018) found reduced growth of *M. peelii* due to bioenergetic constraints resulting from increased flow velocities and lower temperatures directly below impoundments. Other ex situ artificial studies have found similar responses in fish condition due to flow regime disturbance. Puffer et al. (2015) reported a reduction in body fat of Atlantic salmon *Salmo salar* L. 1758 in simulated hydropeaking experiments (with stable flow conditions used as a control) designed to replicate the impacts of river flow regime disturbance as a result of hydroelectric energy production. There were also increased movement rates as a result of hydropeaking, potentially leading to further reductions in the condition (Puffer et al., 2015). The resulting alterations to the flow regime from hydropeaking potentially mirror those currently experienced in the MDB as a result of irrigation flows released from water storages, which most often cause rapid rises and declines in flow magnitude. Contrary to our findings, Paukert and Rogers (2004) found that the condition of flannelmouth suckers (*Catostomus latipinnis* S. F. Baird & Girard, 1853) increased in the Colorado River below a dam that stabilised base flows, potentially increasing productivity as a result of a larger euphotic zone. However, the authors also found strong seasonal variation in the condition and noted that tolerance to hydrological alteration may be species specific (Paukert & Rogers, 2004). Ultimately, the impacts of flow regime disturbance are broad, and it is difficult to attribute a single mechanism by which fish condition is impacted at this spatial scale.

### 4.2 Temporal variation in fish condition

Our basin‐scale analysis uncovered species‐specific temporal trends in fish condition that potentially relate to drought conditions that occurred in 2017–2019 (Bureau of Meteorology, 2020), and the breaking of this drought with above‐average rainfall and increased flows across much of the basin in 2020 and 2021 (Figure S1) (Bureau of Meteorology, 2021; Hladyz et al., 2022). The contrasting hydrological states between these two periods are indicative of the commonly referred to ‘boom and bust’ nature of many large inland temperate rivers such as those within the MDB (King et al., 2003; Sternberg et al., 2012). The impacts these conditions have on the basal resources supporting fish populations could potentially explain the predicted condition of *N. erebi*, *C. carpio* and *M. peelii* (King et al., 2003; Sternberg et al., 2012).

We found a significant reduction in the condition of *N. erebi* in 2020 and 2021, immediately following the breaking of the 2017–2019 drought. We postulate that this sharp decrease in condition following a 3‐year drought is possibly a result of a sudden change in availability of a high‐quality food resource. During extended no‐flow periods, stabilisation of water levels has been shown to cause a ‘bathtub ring’ of algal resources around littoral margins of isolated waterholes (Balcombe et al., 2014; Bunn, Balcombe, et al., 2006; Bunn et al., 2003). *N. erebi* are known to undergo population booms following waterhole isolation as a result of an increase in production of algae (Balcombe et al., 2005; Sternberg et al., 2008). Balcombe et al. (2014) reported that the condition of *N. erebi* increased with time post floodplain inundation. Although not significant, an increase in the condition of *N. erebi* was observed during the 2018 drought, potentially because of increased algal availability. However, following higher flows across the basin in 2020 and 2021, these algal resources were potentially lost, as isolated waterholes returned to a lotic state. In place of algae, *N. erebi* shift diets to lower‐quality allochthonous detritus, which has been shown to be more abundant in its diet during wet periods (Balcombe et al., 2005).

Temporal trends in *M. peelii* and *C. carpio* condition uncovered in our basin‐scale analysis may also relate to the variable climatic conditions experienced over the study period. However, the contrasting temporal trends in the condition of these species compared to *N. erebi* potentially highlight differences in food resources important to each species and how these resources respond to drought. *M. peelii* condition was significantly lower (compared to 2015) both in 2019, the last year of draught, and in 2020, the following non‐drought year, followed by a marked increase in the condition to pre‐drought levels in 2021. Similarly, *C. carpio* condition was significantly lower (compared to 2015) in the last two drought years, followed by an improvement in the condition to pre‐drought levels in 2020 and 2021. An important difference between these two species and *N. erebi* is that both *M. peelii* (an apex carnivore) and *C. carpio* (a mid‐level generalist) occupy a higher trophic position than *N. erebi* (an algivore/detritivore) (Ebner, 2006; Koehn et al., 2020; Lintermans, 2023). In contrast to *N. erebi* that benefit from littoral algal production during periods of drought, food resources supporting *M. peelii* and *C. carpio* are likely to be severely degraded during extended droughts, as productivity generating quality food resources at higher trophic levels is reduced (Balcombe et al., 2005; Sternberg et al., 2012). This reduction in productivity that supports the availability of high‐quality food resources could potentially explain the temporal trends in condition. Differences in the impact of flow regime disturbance on ecosystems containing fish that occupy contrasting trophic niches and positions have been previously documented in the broader literature. Wang et al. (2016) employed stable isotope analysis to quantify changes in trophic niches to hydrological alteration caused by damming of the Yangtze River in China. These authors found changes in the availability of basal resources at inundated sites with lower flow rates, resulting in reduced resource availability and species diversity (Wang et al., 2016).

Strong patterns of temporal variability uncovered through our analysis of condition at the basin scale were not as evident for our river‐valley scale analysis. Although *N. erebi* displayed significantly lower condition after the drought, in line with our basin‐scale results, patterns of condition in *C. carpio* were inverse of those found at the basin scale. *C. carpio* condition was highest during the drought and did not reflect a ‘boom’ in productivity after the drought broke in 2019. Similarly, *M. ambigua* condition was also lower after the drought broke. These findings highlight potential inconsistencies in results that can occur when comparing ecological indicators across multiple spatial scales (Fausch et al., 2002). Our river‐valley scale analysis focuses on one reach, or a small collection of river reaches that when compared to our basin‐scale dataset potentially do not reflect the complete suite of environmental variability that can occur across an entire sub‐basin. Therefore, patterns in condition responses to environmental factors are likely to vary depending on the spatial context of the dataset (Wheatley & Johnson, 2009).

Collectively, our findings and the findings of similar studies are in line with conceptual frameworks that outline the importance of flow regimes and alterations to these regimes on overall freshwater productivity in river environments (Bunn & Arthington, 2002; Humphries et al., 2014; Tockner et al., 2000). Differences in productivity supporting fish condition between ‘boom’ and ‘bust’ states largely relate to flow variability (Bunn, Balcombe, et al., 2006; Tonkin et al., 2011), the main tenet of river productivity paradigms such as the flood pulse concept and extended flood pulse concept (Junk et al., 1989; Tockner et al., 2000).

### 4.3 Size‐specific variation in fish condition

Our study incorporated the effect of fish size on condition by categorising fish into length quartiles, allowing comparisons among size classes, with differences potentially being a result of extrinsic (environmental) and intrinsic (physiological) factors. For all species across both basin and river‐valley extents (with the exception of *C. carpio* within our river‐valley scale analysis), we found generally that larger fish (Q4) were in significantly better condition than smaller fish (Q1) (Figure 8; see Table S2 for length quartile pair‐wise comparisons). The size of a fish has bearing on how it interacts with its environment, including aspects of resource utilisation as well as inter‐ and intraspecific interactions (King, 2005). For example, factors such as swimming performance and gape size generally increase as fish become larger, meaning larger fish can potentially utilise different habitat types and consume higher‐quality food resources (Bassar et al., 2016; Luiz et al., 2019; Stoffels et al., 2020; Whiterod, 2013). In the context of competition between conspecifics and allospecifics, increased body size provides numerous competitive advantages such as greater ability for resource acquisition and displacement of smaller individuals from mutually preferred habitat (Anaya‐Rojas et al., 2021; Young, 2004). These competitive advantages could potentially explain why larger fish in our analysis were generally in better condition than smaller fish.

Size can also be an indicator of the ontogenetic state or life stage of a fish, which has bearing on how a fish allocates energy resources to perform functions related to their life history. In food‐limited environments, trade‐offs between various physiological processes, including growth, reproduction or maintenance of condition, must occur for fish of all sizes; however, where a fish allocates energy is often dependent on life stage (Brosset et al., 2016). In the case of juvenile fish, trade‐offs between gains in condition and growth have been observed. In these instances, increases in juvenile length are prioritised over gains in condition with the benefit of increased survivorship due to lower predation (Karametsidis et al., 2023). Conversely, larger fish with competitive advantages in resource acquisition may be able to attribute a greater proportion of their energy budget to maintaining a higher condition (Kingsbury et al., 2020). Additionally, increases in older fish length will naturally slow down due to physiological limitations; however, increases in girth may continue, resulting in larger fish that are more in better condition (Chen & Liu, 2022).

Anthropogenic disturbances may also disproportionately impact fishes of a specific size (Stoffels et al., 2020). For example, modelled reductions in growth in juvenile *M. peelii* sampled directly below a hypolimnetic‐releasing impoundment were likely a result of high energetic costs associated with flow velocity, and larger *M. peelii* with increased swimming abilities are unlikely to share these bioenergetic consequences (Whiterod, 2013; Whiterod et al., 2018). Increased energetic requirements of smaller fish could potentially reduce condition compared to larger fish that require less energy to moderate the impacts of flow disturbances.

### 4.4 Fish condition across a latitudinal gradient

Our basin‐scale analysis found a strong latitudinal gradient in the condition of *M. peelii*, *M. ambigua* and *C. carpio*. Inferring the mechanistic factors contributing to this trend is difficult, as latitude itself is not what drives condition, but rather it is other factors that potentially change over a latitudinal gradient. Lower productivity, which is characteristic of the NMDB (Balcombe et al., 2012), rivers may ultimately lead to decreased availability of high‐quality food items for fish species that occupy a higher trophic position such as *C. carpio*, *M. ambigua* and *M. peelii*, resulting in decreased condition of these species at more northern latitudes (Balcombe et al., 2005). A latitudinal gradient in condition was not found for *N. erebi*, potentially because a major high‐quality food source for this species persists in isolated waterholes commonly found in the NMDB. Additionally, Wright et al. (2020) found there was a larger proportion of smaller *M. ambigua* in the NMDB, which combined with our finding that smaller fish are in generally poorer condition across the MDB, could explain the latitudinal relationship for this species. Strong latitudinal trends in fish production in the Northern Hemisphere have been observed that mirror our findings. In a study of secondary production‐latitude relationships of multiple fish species across a continental‐scale gradient, Rypel and David (2017) found that numerous species' populations increased in productivity at higher latitudes. These authors cited countergradient growth, defined as faster sub‐annual growth in higher‐latitude fish populations as a compensation mechanism for shorter growing seasons (Rypel, 2012), and increased food availability at higher latitudes as potential reasons for their observed latitudinal trends in productivity (Rypel & David, 2017). Previous studies on spatial growth variability of *M. peelii* and *M. ambigua* have not found strong latitudinal trends, indicating that countergradient growth may be less important for native Australian MDB species (Svozil et al., 2019; Wright et al., 2020). Therefore, the strong latitudinal trend in condition observed in our study may be instead related to food availability or other factors related to increased productivity at more southern latitudes.

### 4.5 Responses to antecedent flow metrics at a river‐valley scale

At a river‐valley scale, we found that condition increased with increasing mean antecedent flow magnitude for *C. carpio*, *M. ambigua* and *M. peelii*. This flow metric gives insight into the hydrological conditions a fish has experienced in the previous 365 days. Increasing flow magnitude is potentially associated with increasing rates of productivity, with potential implications hypothesised to be improved fish condition due to greater and richer food resources (Balcombe et al., 2012). Our finding is in line with previous studies investigating the impacts of antecedent flow magnitude, such as that by Balcombe et al. (2014), who found an increase in the condition of *M. ambigua* as daily discharge increased in a 3‐month antecedent period. Furthermore, decreased flow magnitudes as a result of river regulation have also been shown to impact fish growth. For example, Tonkin et al. (2017) assessed the impact of flow components such as magnitude on *M. peelii* and trout cod *Maccullochella macquariensis* Cuvier 1829 growth in the Murray River. Although improved growth doesn't necessarily relate to improved condition, our findings and the findings of Tonkin et al. (2017) emphasise the detrimental impact of river regulation on somatic indices of fish in floodplain rivers, both in the MDB and throughout the world (Tonkin et al., 2017).

Our river‐valley scale analysis also found that the number of high‐flow days (above the 90th percentile of flow magnitude) in the 365 days leading up to fish capture had a significant negative impact on the condition of *N. erebi*. This finding is in line with our hypothesis that increased flow can potentially remove a valuable algal food resource for *N. erebi* that occurs in no‐flow environments. Additionally, other studies have shown that prolonged periods of high flows cause reductions in fish condition, potentially resulting from fish experiencing higher energy costs associated with swimming and feeding (Luz‐Agostinho et al., 2009; Petenuci et al., 2016).

River ecosystem management can be dynamic and targeted, for example, releasing environmental water to support specific life‐history traits of fishes and other organisms, or more passive and indirect, such as improving overall ecosystem integrity through removal of water infrastructure such as weirs and levees. Our river‐valley analysis suggests that site‐specific flow conditions may have a greater influence on fish condition than broader basin‐scale disturbances, as although FRDI was included in the global model for our river‐valley scale analysis, it was not selected in the most parsimonious model for all species at this scale. Consequently, management practices that focus on local conditions and tailored interventions, such as adjusting environmental flow releases, could be more effective in improving fish condition (Hladyz et al., 2022; Stuart et al., 2019). This finding is contrary to contemporary flow management strategies that endeavour to take a basin‐wide approach to environmental flow management (Stewardson & Guarino, 2018). Although basin‐scale management strategies are valuable for considering the interconnectivity between river systems, recognising the dominant role of local drivers will help managers to develop more precise and effective strategies that address the specific needs of fish populations in different river valleys, ultimately enhancing conservation efforts and ecological outcomes (Wang et al., 2006).

### 4.6 River‐valley differences in fish condition

We found a high degree of variability in the condition of each species among individual river valleys (Figure 10). Generally, no clear pattern of fish condition was apparent among species and rivers, apart from consistently lower condition values for *C. carpio*, *M. ambigua* and *M. peelii* in the Murrumbidgee River. This finding contradicts previous reports on condition of *N. erebi*, *M. peelii* and *M. ambigua* in the same study areas, where little spatial variation in condition was observed (Stoffels et al., 2016). However, the conditions in 2014–2015 when the study was undertaken were much different, with severe hydrological events occurring after this time that likely resulted in increased inter‐river variability in fish condition. Notably, significant fish kill events occurred in the lower Darling River between 2018 and 2020 that would have likely impacted fish condition during the study period within this region (Sheldon et al., 2021; Stocks et al., 2021). Although the current study employed metrics in both basin‐ and valley‐scale analyses that potentially incorporate conditions contributing to fish kill events (e.g., flow and temperature variability), we found limited evidence of changes in fish condition that could be implicated as a result of isolated fish kill events such as those occurring in the lower Darling River. It has been previously recognised that drivers of condition in fish can be river specific (Balcombe et al., 2014), and our findings somewhat support that hypothesis.

## 5 CONCLUSION

Anthropogenic influences and their resulting environmental alterations in freshwater ecosystems certainly affect the condition of fishes. We assessed the impact of hydrological disturbance on the conditions of *N. erebi*, *C. carpio*, *M. ambigua* and *M. peelii* to uncover responses to altered flow regimes and elucidate potential underlying ecological mechanisms. In general, fish condition was sensitive to both broad‐ and fine‐scale metrics; however, the direction and magnitude of these relationships were complex, likely owing to species‐ and scale‐specific interactions. At the basin scale, disturbances to the flow regime, caused by water infrastructure such as dams and levees, had a negative impact on the condition of *M. peelii* and *M. ambigua*, likely due to alterations in food‐web dynamics and resource availability. We also observed trends in condition potentially related to ‘boom and bust’ productivity dynamics commonly observed in large inland temperate rivers. Our river‐valley scale results indicating significant impacts of antecedent flow variables can guide targeted local interventions, such as optimising environmental flow releases to enhance fish condition. Altogether, our results suggest that drivers of body condition in fishes of the MDB are scale dependent and species specific, and that future studies of drivers of condition in freshwater ecosystems should consider the context of scale, and how this relates to disturbances in question. Our findings support the growing body of evidence of the negative effects of hydrological disturbance manifested at the individual organism level, with the effects predicted to be exacerbated by further development of water infrastructure (in some regions) and climate change.

## AUTHOR CONTRIBUTIONS

Study conception and design: Maxwell C. Mallett, Mark J. Kennard, Jason D. Thiem and Gavin L. Butler. Data collection: Maxwell C. Mallett, Mark J. Kennard and Luke Carpenter‐Bundhoo. Analysis and interpretation of results: Maxwell C. Mallett, Mark J. Kennard, Jason D. Thiem, Luke Carpenter‐Bundhoo and Gavin L. Butler. Draft manuscript preparation: Maxwell C. Mallett, Mark J. Kennard, Jason D. Thiem, Luke Carpenter‐Bundhoo and Gavin L. Butler. All authors reviewed the results and approved the final version of the manuscript.

## FUNDING INFORMATION

This work was supported by an Australian Government Research Training Program Scholarship, a New South Wales Department of Primary Industries (Fisheries) top‐up scholarship and the Barry Jonassen Award from the Australian Society for Fish Biology.

## Supporting information


**Data S1.** Supporting information.

## Data Availability

The data that support the findings of this study are available in Commonwealth Environmental Water Holder (CEWH) Flow‐MER Program online database at https://data.gov.au/data/dataset/flow-mer-fish-length-weight.

## References

[jfb70033-bib-0001] Allan, D. , Erickson, D. , & Fay, J. (1997). The influence of catchment land use on stream integrity across multiple spatial scales. Freshwater Biology, 37(1), 149–161. 10.1046/j.1365-2427.1997.d01-546.x

[jfb70033-bib-0002] Anaya‐Rojas, J. M. , Bassar, R. D. , Potter, T. , Blanchette, A. , Callahan, S. , Framstead, N. , Reznick, D. , & Travis, J. (2021). The evolution of size‐dependent competitive interactions promotes species coexistence. Journal of Animal Ecology, 90(11), 2704–2717. 10.1111/1365-2656.13577 34389988

[jfb70033-bib-0003] Anderson, J. R. , Morison, A. , & Ray, D. J. (1992). Validation of the use of thin‐sectioned otoliths for determining the age and growth of Golden perch, Macquaria ambigua (Perciformes: Percichthyidae), in the lower Murray‐Darling basin, Australia. Marine and Freshwater Research, 43(5), 1103. 10.1071/MF9921103

[jfb70033-bib-0004] Arthington, A. H. , Bunn, S. E. , Poff, N. L. , & Naiman, R. J. (2006). The challenge of providing environmental flow rules to sustain river ecosystems. Ecological Applications, 16(4), 1311–1318. 10.1890/1051-0761(2006)016[1311:tcopef]2.0.co;2 16937799

[jfb70033-bib-0005] Balcombe, S. R. , Arthington, A. H. , & Sternberg, D. (2014). Fish body condition and recruitment responses to antecedent flows in dryland rivers are species and river specific. River Research and Applications, 30(10), 1257–1268. 10.1002/rra.2797

[jfb70033-bib-0006] Balcombe, S. R. , Bunn, S. E. , McKenzie‐Smith, F. J. , & Davies, P. M. (2005). Variability of fish diets between dry and flood periods in an arid zone floodplain river. Journal of Fish Biology, 67(6), 1552–1567. 10.1111/j.1095-8649.2005.00858.x

[jfb70033-bib-0007] Balcombe, S. R. , Lobegeiger, J. S. , Marshall, S. M. , Marshall, J. C. , Ly, D. , & Jones, D. N. (2012). Fish body condition and recruitment success reflect antecedent flows in an Australian dryland river. Fisheries Science, 78(4), 841–847. 10.1007/s12562-012-0519-z

[jfb70033-bib-0008] Barton, K. , & Barton, M. K. (2015). Package ‘mumin’. In Version.

[jfb70033-bib-0009] Bassar, R. D. , Childs, D. Z. , Rees, M. , Tuljapurkar, S. , Reznick, D. N. , & Coulson, T. (2016). The effects of asymmetric competition on the life history of Trinidadian guppies. Ecology Letters, 19(3), 268–278. 10.1111/ele.12563 26843397 PMC4991285

[jfb70033-bib-0010] Baumgartner, L. J. (2007). Diet and feeding habits of predatory fishes upstream and downstream of a low‐level weir. Journal of Fish Biology, 70(3), 879–894. 10.1111/j.1095-8649.2007.01352.x

[jfb70033-bib-0011] Bice, C. , Zampatti, B. , & Tonkin, Z. (2016). The influence of weir pool raising in the South Australian lower River Murray on condition and growth of Australian smelt (Retropinna semoni). *SARDI Research Report Series‐South Australian Research and Development Institute*(907).

[jfb70033-bib-0012] Blackwell, B. G. , Brown, M. L. , & Willis, D. W. (2000). Relative weight (Wr) status and current use in fisheries assessment and management. Reviews in Fisheries Science, 8(1), 1–44. 10.1080/10641260091129161

[jfb70033-bib-0013] Brosset, P. , Averty, A. , Mathieu‐Resuge, M. , Schull, Q. , Soudant, P. , & Lebigre, C. (2023). Fish morphometric body condition indices reflect energy reserves but other physiological processes matter. Ecological Indicators, 154(110), 860. 10.1016/j.ecolind.2023.110860

[jfb70033-bib-0014] Brosset, P. , Lloret, J. , Muñoz, M. , Fauvel, C. , Van Beveren, E. , Marques, V. , Fromentin, J.‐M. , Ménard, F. , & Saraux, C. (2016). Body reserves mediate trade‐offs between life‐history traits: New insights from small pelagic fish reproduction. Royal Society open Science, 3(10), 160–202. 10.1098/rsos.160202 PMC5098963 27853538

[jfb70033-bib-0015] Bunn, S. E. , & Arthington, A. H. (2002). Basic principles and ecological consequences of altered flow regimes for aquatic biodiversity. Environmental Management, 30(4), 492–507. 10.1007/s00267-002-2737-0 12481916

[jfb70033-bib-0016] Bunn, S. E. , Balcombe, S. R. , Davies, P. M. , Fellows, C. S. , & McKenzie‐Smith, F. J. (2006). Aquatic productivity and food webs of desert river ecosystems. In Ecology of Desert Rivers (pp. 76–99). Cambridge University Press.

[jfb70033-bib-0017] Bunn, S. E. , Davies, P. M. , & Winning, M. (2003). Sources of organic carbon supporting the food web of an arid zone floodplain river. Freshwater Biology, 48(4), 619–635. 10.1046/j.1365-2427.2003.01031.x

[jfb70033-bib-0018] Bunn, S. E. , Thoms, M. C. , Hamilton, S. K. , & Capon, S. J. (2006). Flow variability in dryland rivers: Boom, bust and the bits in between. River Research and Applications, 22(2), 179–186. 10.1002/rra.904

[jfb70033-bib-0019] Bureau of Meteorology . (2010). Australian hydrological geospatial fabric (geofabric) product guide. Bureau of Meteorology.

[jfb70033-bib-0020] Bureau of Meteorology . (2020). Special Climate Statement 70 update: drought conditions in Australia and impact on water resources in the Murray‐Darling Basin .

[jfb70033-bib-0021] Bureau of Meteorology . (2021). Australia in the 2020 to 2021 financial year. Bureau of Meteorology. http://www.bom.gov.au/climate/updates/articles/a039.shtml

[jfb70033-bib-0022] Butler, G. L. , Davis, T. R. , Brooks, S. G. , Bowen, C. , Cameron, L. M. , Rowland, S. J. , Smith, D. , St Vincent Welch, J. , & Carpenter‐Bundhoo, L. (2022). Combining bio‐telemetry and underwater imagery to elucidate the reproductive behaviour of a large, long‐lived Australian freshwater teleost. Journal of Environmental Management, 317, 115298. 10.1016/j.jenvman.2022.115298 35617858

[jfb70033-bib-0023] CEWH . (2023). Fish length weight. Flow‐MER Program . https://data.gov.au/data/dataset/flow-mer-fish-length-weight

[jfb70033-bib-0024] Chen, X. , & Liu, B. (2022). Life history and early development of fishes. In X. Chen & B. Liu (Eds.), Biology of fishery resources (pp. 55–69). Springer Nature. 10.1007/978-981-16-6948-4_3

[jfb70033-bib-0025] Cruz, D. O. , Kingsford, R. T. , Suthers, I. M. , Rayner, T. S. , Smith, J. A. , & Arthington, A. H. (2020). Connectivity but not recruitment: Response of the fish community to a large‐scale flood on a heavily regulated floodplain. Ecohydrology, 13(3), e2194. 10.1002/eco.2194

[jfb70033-bib-0026] Davies, B. , Snaddon, C. , Wishart, M. , Thoms, M. , & Meador, M. (2000). A biogeographical approach to interbasin water transfers: Implications for river conservation. Global Perspectives on River Conservation: Science, Policy and Practice, 1, 431–444.

[jfb70033-bib-0027] Ebner, B. (2006). Murray cod an apex predator in the Murray River, Australia. Ecology of Freshwater Fish, 15(4), 510–520. 10.1111/j.1600-0633.2006.00191.x

[jfb70033-bib-0028] Fausch, K. D. , Torgersen, C. E. , Baxter, C. V. , & Li, H. W. (2002). Landscapes to riverscapes: Bridging the gap between research and conservation of stream fishes: A continuous view of the river is needed to understand how processes interacting among scales set the context for stream fishes and their habitat. Bioscience, 52(6), 483–498. 10.1641/0006-3568(2002)052[0483:LTRBTG]2.0.CO;2

[jfb70033-bib-0029] Gibbins, C. , Jeffries, M. , & Soulsby, C. (2000). Impacts of an inter‐basin water transfer: Distribution and abundance of Micronecta poweri (Insecta: Corixidae) in the river Wear, north‐east England. Aquatic Conservation: Marine and Freshwater Ecosystems, 10(2), 103–115.

[jfb70033-bib-0030] Hayes, J. , & Shonkwiler, J. (2001). Morphometric indicators of body condition: Worthwhile or wishful think. In Body composition analysis of animals: A handbook of non‐destructive methods (pp. 8–38). Cambridge University Press.

[jfb70033-bib-0031] Hewitt, J. E. , Thrush, S. F. , & Lundquist, C. (2010). Scale‐dependence in ecological systems. Encyclopedia of Life Sciences, 1, 1–7.

[jfb70033-bib-0032] Hladyz, S. , Baumgartner, L. , Bice, C. , Butler, G. , Fanson, B. , Giatas, G. , Koster, W. , Lyon, J. , Stuart, I. , Thiem, J. , Tonkin, Z. , Ye, Q. , Yen, J. , & Zampatti, B. (2022). Basin‐scale evaluation of 2020–21 commonwealth environmental water: Fish. Commonwealth Environmental Water Office (CEWO).

[jfb70033-bib-0033] Humphries, P. (2005). Spawning time and early life history of Murray cod, Maccullochella peelii (Mitchell) in an Australian river. Environmental Biology of Fishes, 72(4), 393–407. 10.1007/s10641-004-2596-z

[jfb70033-bib-0034] Humphries, P. , Keckeis, H. , & Finlayson, B. (2014). The river wave concept: Integrating river ecosystem models. Bioscience, 64(10), 870–882.

[jfb70033-bib-0035] Humphries, P. , King, A. J. , & Koehn, J. D. (1999). Fish, flows and Flood Plains: Links between freshwater fishes and their environment in the Murray‐Darling river system. Australia. Environmental Biology of Fishes, 56(1), 129–151. 10.1023/A:1007536009916

[jfb70033-bib-0036] Jager, H. I. , Chandler, J. A. , Lepla, K. B. , & Van Winkle, W. (2001). A theoretical study of river fragmentation by dams and its effects on white sturgeon populations. Environmental Biology of Fishes, 60, 347–361.

[jfb70033-bib-0037] Junk, W. J. , Bayley, P. B. , & Sparks, R. E. (1989). The flood pulse concept in river‐floodplain systems. Canadian Special Publication Fisheries and Aquatic Sciences, 106, 110–127.

[jfb70033-bib-0038] Karametsidis, G. , Rueda, L. , Bellido, J. M. , Esteban, A. , García, E. , de Gil Sola, L. , Pennino, M. G. , Pérez‐Gil, J. L. , & Hidalgo, M. (2023). The trade‐off between condition and growth shapes juveniles' survival of harvested demersal fish of the Mediterranean sea. Marine Environmental Research, 184, 105–844. 10.1016/j.marenvres.2022.105844 36603343

[jfb70033-bib-0039] Kaufman, S. , Johnston, T. , Leggett, W. , Moles, M. , Casselman, J. , & Schulte‐Hostedde, A. (2007). Relationships between body condition indices and proximate composition in adult walleyes. Transactions of the American Fisheries Society, 136(6), 1566–1576. 10.1577/T06-262.1

[jfb70033-bib-0040] Kelly, V. J. (2001). Influence of reservoirs on solute transport: A regional‐scale approach. Hydrological Processes, 15(7), 1227–1249.

[jfb70033-bib-0041] Kennard, M. J. , Olden, J. D. , Arthington, A. H. , Pusey, B. J. , & Poff, N. L. (2007). Multiscale effects of flow regime and habitat and their interaction on fish assemblage structure in eastern Australia. Canadian Journal of Fisheries and Aquatic Sciences, 64(10), 1346–1359. 10.1139/f07-108

[jfb70033-bib-0042] King, A. J. (2005). Ontogenetic dietary shifts of fishes in an Australian floodplain river. Marine and Freshwater Research, 56(2), 215–225. 10.1071/MF04117

[jfb70033-bib-0043] King, A. J. , Humphries, P. , & Lake, P. S. (2003). Fish recruitment on floodplains: The roles of patterns of flooding and life history characteristics. Canadian Journal of Fisheries and Aquatic Sciences, 60(7), 773–786. 10.1139/f03-057

[jfb70033-bib-0044] King, A. J. , Tonkin, Z. , & Mahoney, J. (2009). Environmental flow enhances native fish spawning and recruitment in the Murray River. Australia. River Research and Applications, 25(10), 1205–1218.

[jfb70033-bib-0045] Kingsbury, K. M. , Gillanders, B. M. , Booth, D. J. , Coni, E. O. C. , & Nagelkerken, I. (2020). Range‐extending coral reef fishes trade‐off growth for maintenance of body condition in cooler waters. Science of the Total Environment, 703(134), 598. 10.1016/j.scitotenv.2019.134598 31767323

[jfb70033-bib-0046] Knox, R. L. , Wohl, E. E. , & Morrison, R. R. (2022). Levees don't protect, they disconnect: A critical review of how artificial levees impact floodplain functions. Science of the total environment, 837, 155773. 10.1016/j.scitotenv.2022.155773 35537517

[jfb70033-bib-0047] Koehn, J. D. , Balcombe, S. R. , & Zampatti, B. P. (2019). Fish and flow management in the Murray–Darling basin: Directions for research. Ecological Management & Restoration, 20(2), 142–150.

[jfb70033-bib-0048] Koehn, J. D. , Copeland, C. , & Stamation, K. (2014). The future for managing fishes in the Murray‐Darling basin, south‐eastern Australia. Ecological Management & Restoration, 15(s1), 1–2. 10.1111/emr.12098

[jfb70033-bib-0049] Koehn, J. D. , Raymond, S. M. , Stuart, I. , Todd, C. R. , Balcombe, S. R. , Zampatti, B. P. , Bamford, H. , Ingram, B. A. , Bice, C. M. , & Burndred, K. (2020). A compendium of ecological knowledge for restoration of freshwater fishes in Australia's Murray–Darling basin. Marine and Freshwater Research, 71(11), 1391–1463.

[jfb70033-bib-0050] Kopf, S. M. , Humphries, P. , & Watts, R. J. (2014). Ontogeny of critical and prolonged swimming performance for the larvae of six Australian freshwater fish species. Journal of Fish Biology, 84(6), 1820–1841. 10.1111/jfb.12399 24814314

[jfb70033-bib-0051] Lintermans, M. (2023). Fishes of the Murray‐Darling basin (2nd ed.). Australian River Restoration Centre.

[jfb70033-bib-0052] Lowe, W. H. , Likens, G. E. , & Power, M. E. (2006). Linking scales in stream ecology. Bioscience, 56(7), 591–597. 10.1641/0006-3568(2006)56[591:LSISE]2.0.CO;2

[jfb70033-bib-0053] Luiz, O. J. , Crook, D. A. , Kennard, M. J. , Olden, J. D. , Saunders, T. M. , Douglas, M. M. , Wedd, D. , & King, A. J. (2019). Does a bigger mouth make you fatter? Linking intraspecific gape variability to body condition of a tropical predatory fish. Oecologia, 191(3), 579–585. 10.1007/s00442-019-04522-w 31583451

[jfb70033-bib-0054] Luz‐Agostinho, K. , Agostinho, A. , Gomes, L. , Julio, H. F. , & Fugi, R. (2009). Effects of flooding regime on the feeding activity and body condition of piscivorous fish in the upper Paraná River floodplain. Brazilian Journal of Biology, 69, 481–490. 10.1590/s1519-69842009000300004 19738956

[jfb70033-bib-0055] Magoulick, D. D. , & Kobza, R. M. (2003). The role of refugia for fishes during drought: A review and synthesis. Freshwater Biology, 48(7), 1186–1198. 10.1046/j.1365-2427.2003.01089.x

[jfb70033-bib-0056] Mallett, M. C. , Thiem, J. D. , Butler, G. L. , & Kennard, M. J. (2024). A systematic review of approaches to assess fish health responses to anthropogenic threats in freshwater ecosystems. Conservation Physiology, 12(1), coae022. 10.1093/conphys/coae022 38706739 PMC11069195

[jfb70033-bib-0057] Milot, E. , Cohen, A. A. , Vézina, F. , Buehler, D. M. , Matson, K. D. , & Piersma, T. (2014). A novel integrative method for measuring body condition in ecological studies based on physiological dysregulation. Methods in Ecology and Evolution, 5(2), 146–155. 10.1111/2041-210X.12145

[jfb70033-bib-0058] Nakagawa, S. , & Schielzeth, H. (2013). A general and simple method for obtaining R2 from generalized linear mixed‐effects models. Methods Ecol Evol, 4(2), 133–142.

[jfb70033-bib-0059] Newbery, B. , Connolly, R. M. , Melvin, S. D. , & Sievers, M. (2024). The utility of non‐lethal morphometrics to evaluate fish condition. Austral Ecology, 49(3), e13510. 10.1111/aec.13510

[jfb70033-bib-0060] Paukert, C. , & Rogers, R. S. (2004). Factors affecting condition of Flannelmouth suckers in the Colorado River, grand canyon, Arizona. North American Journal of Fisheries Management, 24(2), 648–653. 10.1577/M03-087.1

[jfb70033-bib-0061] Petenuci, M. E. , Rocha, I. d. N. A. , de Sousa, S. C. , Schneider, V. V. A. , da Costa, L. A. M. A. , & Visentainer, J. V. (2016). Seasonal variations in lipid content, fatty acid composition and nutritional profiles of five freshwater fish from the Amazon Basin. Journal of the American Oil Chemists' Society, 93(10), 1373–1381. 10.1007/s11746-016-2884-8

[jfb70033-bib-0062] Pope, K. L. , & Kruse, C. G. (2007). Condition. In C. S. Guy & M. L. Brown (Eds.), Analysis and interpretation of freshwater fisheries data (pp. 423–471). American Fisheries Society. 10.47886/9781888569773

[jfb70033-bib-0063] Puffer, M. , Berg, O. K. , Huusko, A. , Vehanen, T. , Forseth, T. , & Einum, S. (2015). Seasonal effects of hydropeaking on growth, energetics and movement of juvenile Atlantic Salmon (Salmo Salar). River Research and Applications, 31(9), 1101–1108. 10.1002/rra.2801

[jfb70033-bib-0064] Raupach, M. , Kirby, J. , Barrett, D. , & Briggs, P. (2001). Balances of water, carbon, nitrogen and phosphorus in Australian landscapes:(1) Project description and results (CSIRO Land and Water Technical Report 40/01, Issue.

[jfb70033-bib-0065] Richardson, S. M. , Hanson, J. M. , & Locke, A. (2002). Effects of impoundment and water‐level fluctuations on macrophyte and macroinvertebrate communities of a dammed tidal river. Aquatic Ecology, 36(4), 493–510. 10.1023/A:1021137630654

[jfb70033-bib-0066] Rolls, R. J. , Wolfenden, B. , Heino, J. , Butler, G. L. , & Thiem, J. D. (2023). Scale dependency in fish beta diversity–hydrology linkages in lowland rivers. Journal of Biogeography, 50(10), 1692–1709. 10.1111/jbi.14672

[jfb70033-bib-0067] Rose, K. A. , Sable, S. , DeAngelis, D. L. , Yurek, S. , Trexler, J. C. , Graf, W. , & Reed, D. J. (2015). Proposed best modeling practices for assessing the effects of ecosystem restoration on fish. Ecological Modelling, 300, 12–29. 10.1016/j.ecolmodel.2014.12.020

[jfb70033-bib-0068] Rypel, A. L. (2012). Meta‐analysis of growth rates for a circumpolar fish, the northern pike (Esox lucius), with emphasis on effects of continent, climate and latitude. Ecology of Freshwater Fish, 21(4), 521–532.

[jfb70033-bib-0098] Rypel, A. L. , & David, S. R. (2017). Pattern and scale in latitude–production relationships for freshwater fishes. Ecosphere, 8(1). e01660. 10.1002/ecs2.1660

[jfb70033-bib-0069] Schlosser, I. J. (1985). Flow regime, juvenile abundance, and the assemblage structure of stream fishes. Ecology, 66(5), 1484–1490. 10.2307/1938011

[jfb70033-bib-0070] Sheldon, F. , Barma, D. , Baumgartner, L. , Bond, N. , Mitrovic, S. , & Vertessy, R. (2021). Assessment of the causes and solutions to the significant 2018–19 fish deaths in the lower Darling River, New South Wales, Australia. Marine and Freshwater Research, 73(2), 147–158.

[jfb70033-bib-0071] Stein, J. , Hutchinson, M. , & Stein, J. (2014). A new stream and nested catchment framework for Australia. Hydrology and Earth System Sciences, 18(5), 1917–1933.

[jfb70033-bib-0072] Stein, J. , Stein, J. , & Nix, H. (2002). Spatial analysis of anthropogenic river disturbance at regional and continental scales: Identifying the wild rivers of Australia. Landscape and Urban Planning, 60(1), 1–25. 10.1016/S0169-2046(02)00048-8

[jfb70033-bib-0074] Sternberg, D. , Balcombe, S. R. , Marshall, J. C. , Lobegeiger, J. S. , & Arthington, A. H. (2012). Subtle ‘boom and bust’ response of Macquaria ambigua to flooding in an Australian dryland river. Environmental Biology of Fishes, 93(1), 95–104. 10.1007/s10641-011-9895-y

[jfb70033-bib-0073] Sternberg, D. , Balcombe, S. , Marshall, J. , & Lobegeiger, J. (2008). Food resource variability in an Australian dryland river: Evidence from the diet of two generalist native fish species. Marine and Freshwater Research, 59(2), 137–144.

[jfb70033-bib-0076] Stewardson, M. J. , & Guarino, F. (2018). Basin‐scale environmental water delivery in the Murray–Darling, Australia: A hydrological perspective. Freshwater Biology, 63(8), 969–985. 10.1111/fwb.13102

[jfb70033-bib-0075] Stewardson, M. , Walker, G. , & Coleman, M. (2020). Hydrology of the Murray–Darling basin. In B. Hart , N. Byron , N. Bond , C. Pollino , & M. Stewardson (Eds.), Murray‐Darling basin, Australia: Its future management (Vol. 1, pp. 47–73). Elsevier Inc. 10.1016/B978-0-12-818152-2.00003-6

[jfb70033-bib-0077] Stocks, J. R. , Ellis, I. M. , van der Meulen, D. E. , Doyle, J. I. , & Cheshire, K. J. (2021). Kills in the Darling: Assessing the impact of the 2018–20 mass fish kills on the fish communities of the lower Darling–Baaka River, a large lowland river of south‐eastern Australia. Marine and Freshwater Research, 73(2), 159–177.

[jfb70033-bib-0080] Stoffels, R. J. , Weatherman, K. E. , Bond, N. R. , Morrongiello, J. R. , Thiem, J. D. , Butler, G. , Koster, W. , Kopf, R. K. , McCasker, N. , Ye, Q. , Zampatti, B. , & Broadhurst, B. (2020). Stage‐dependent effects of river flow and temperature regimes on the growth dynamics of an apex predator. Global Change Biology, 26(12), 6880–6894. 10.1111/gcb.15363 32970901

[jfb70033-bib-0078] Stoffels, R. , Bond, N. , Weatherman, K. , & Gawne, B. (2016). *2014–15 basin‐scale evaluation of commonwealth environmental water — Fish. Final report prepared for the commonwealth environmental water office by the Murray–Darling freshwater research Centre, MDFRC*. M. D. F. R. Centre.

[jfb70033-bib-0079] Stoffels, R. , Stratford, D. , Bond, N. , & Hale, J. (2018). 2016–17 basin‐scale evaluation of commonwealth environmental water – Fish. La Trobe University.

[jfb70033-bib-0081] Stuart, I. , Sharpe, C. , Stanislawski, K. , Parker, A. , & Mallen‐Cooper, M. (2019). From an irrigation system to an ecological asset: Adding environmental flows establishes recovery of a threatened fish species. Marine and Freshwater Research, 70(9), 1295–1306.

[jfb70033-bib-0082] Svozil, D. P. , Kopf, R. K. , Watts, R. J. , & Nicholls, A. O. (2019). Temperature‐dependent larval survival and growth differences among populations of Murray cod ( Maccullochella peelii). Marine and Freshwater Research, 70(4), 459–468. 10.1071/MF18178

[jfb70033-bib-0083] Tockner, K. , Malard, F. , & Ward, J. (2000). An extension of the flood pulse concept. Hydrological Processes, 14(16–17), 2861–2883.

[jfb70033-bib-0086] Tonkin, Z. D. , King, A. J. , Robertson, A. I. , & Ramsey, D. S. L. (2011). Early fish growth varies in response to components of the flow regime in a temperate floodplain river. Freshwater Biology, 56(9), 1769–1782. 10.1111/j.1365-2427.2011.02612.x

[jfb70033-bib-0084] Tonkin, Z. , Kitchingman, A. , Lyon, J. , Kearns, J. , Hackett, G. , O'Mahony, J. , Moloney, P. D. , Krusic‐Golub, K. , & Bird, T. (2017). Flow magnitude and variability influence growth of two freshwater fish species in a large regulated floodplain river. Hydrobiologia, 797(1), 289–301.

[jfb70033-bib-0085] Tonkin, Z. , Yen, J. , Lyon, J. , Kitchingman, A. , Koehn, J. D. , Koster, W. M. , Lieschke, J. , Raymond, S. , Sharley, J. , Stuart, I. , & Todd, C. (2021). Linking flow attributes to recruitment to inform water management for an Australian freshwater fish with an equilibrium life‐history strategy. Science of the total environment, 752, 141863. 10.1016/j.scitotenv.2020.141863 32889283

[jfb70033-bib-0087] Vila‐Gispert, A. , & Moreno‐Amich, R. (2001). Fish condition analysis by a weighted least squares procedure: Testing geographical differences of an endangered Iberian cyprinodontid. Journal of Fish Biology, 58(6), 1658–1666.

[jfb70033-bib-0088] Wang, J. , Li, L. , Xu, J. , & Gu, B. (2016). Initial response of fish trophic niche to hydrological alteration in the upstream of three gorges dam. Ecological Processes, 5(1), 11. 10.1186/s13717-016-0055-3

[jfb70033-bib-0089] Wang, L. , Seelbach, P. W. , & Lyons, J. (2006). Effects of levels of human disturbance on the influence of catchment, riparian, and reach‐scale factors on fish assemblages. American Fisheries Society Symposium, 48(48), 641–664.

[jfb70033-bib-0090] Warfe, D. M. , Pettit, N. E. , Davies, P. M. , Pusey, B. J. , Hamilton, S. K. , Kennard, M. J. , Townsend, S. A. , Bayliss, P. , Ward, D. P. , Douglas, M. M. , Burford, M. A. , Finn, M. , Bunn, S. E. , & Halliday, I. A. (2011). The ‘wet–dry’ in the wet–dry tropics drives river ecosystem structure and processes in northern Australia. Freshwater Biology, 56(11), 2169–2195. 10.1111/j.1365-2427.2011.02660.x

[jfb70033-bib-0091] Wheatley, M. , & Johnson, C. (2009). Factors limiting our understanding of ecological scale. Ecological Complexity, 6(2), 150–159. 10.1016/j.ecocom.2008.10.011

[jfb70033-bib-0092] Whiterod, N. S. (2013). The swimming capacity of juvenile Murray cod (Maccullochella peelii): An ambush predator endemic to the Murray‐Darling basin, Australia. Ecology of Freshwater Fish, 22(1), 117–126. 10.1111/eff.12009

[jfb70033-bib-0093] Whiterod, N. S. , Meredith, S. N. , Humphries, P. , Sherman, B. S. , Koehn, J. D. , Watts, R. J. , Ingram, B. A. , & Ryan, T. (2018). Flow alteration and thermal pollution depress modelled growth rates of an iconic riverine fish, the Murray cod Maccullochella peelii. Ecology of Freshwater Fish, 27(3), 686–698. 10.1111/eff.12384

[jfb70033-bib-0094] Winemiller, K. O. , & Rose, K. A. (1992). Patterns of life‐history diversification in north American fishes: Implications for population regulation. Canadian Journal of Fisheries and Aquatic Sciences, 49(10), 2196–2218.

[jfb70033-bib-0095] Wright, D. W. , Zampatti, B. P. , Baumgartner, L. J. , Brooks, S. , Butler, G. L. , Crook, D. A. , Fanson, B. G. , Koster, W. , Lyon, J. , Strawbridge, A. , Tonkin, Z. , & Thiem, J. D. (2020). Size, growth and mortality of riverine golden perch (Macquaria ambigua) across a latitudinal gradient. Marine and Freshwater Research, 71(12), 1651–1661.

[jfb70033-bib-0096] Young, K. A. (2004). Asymmetric competition, habitat selection, and niche overlap in juvenile salmonids. Ecology, 85(1), 134–149.

[jfb70033-bib-0097] Zuur, A. F. , Ieno, E. N. , & Elphick, C. S. (2010). A protocol for data exploration to avoid common statistical problems. Methods in Ecology and Evolution, 1(1), 3–14. 10.1111/j.2041-210X.2009.00001.x

