# Supplementary material for: Freshwater fish condition responses to hydrological disturbance are species‐ and scale dependent
Source: J Fish Biol. 2025 Apr 11;107(2):493–510. doi: 10.1111/jfb.70033 (PMC12360142; doi:10.1111/jfb.70033)
Supplement: Supplementary file 1 — Data S1. Supporting information. [file JFB-107-493-s001.docx]

Freshwater fish condition responses to hydrological disturbance are species and scale dependent – supplementary material

**
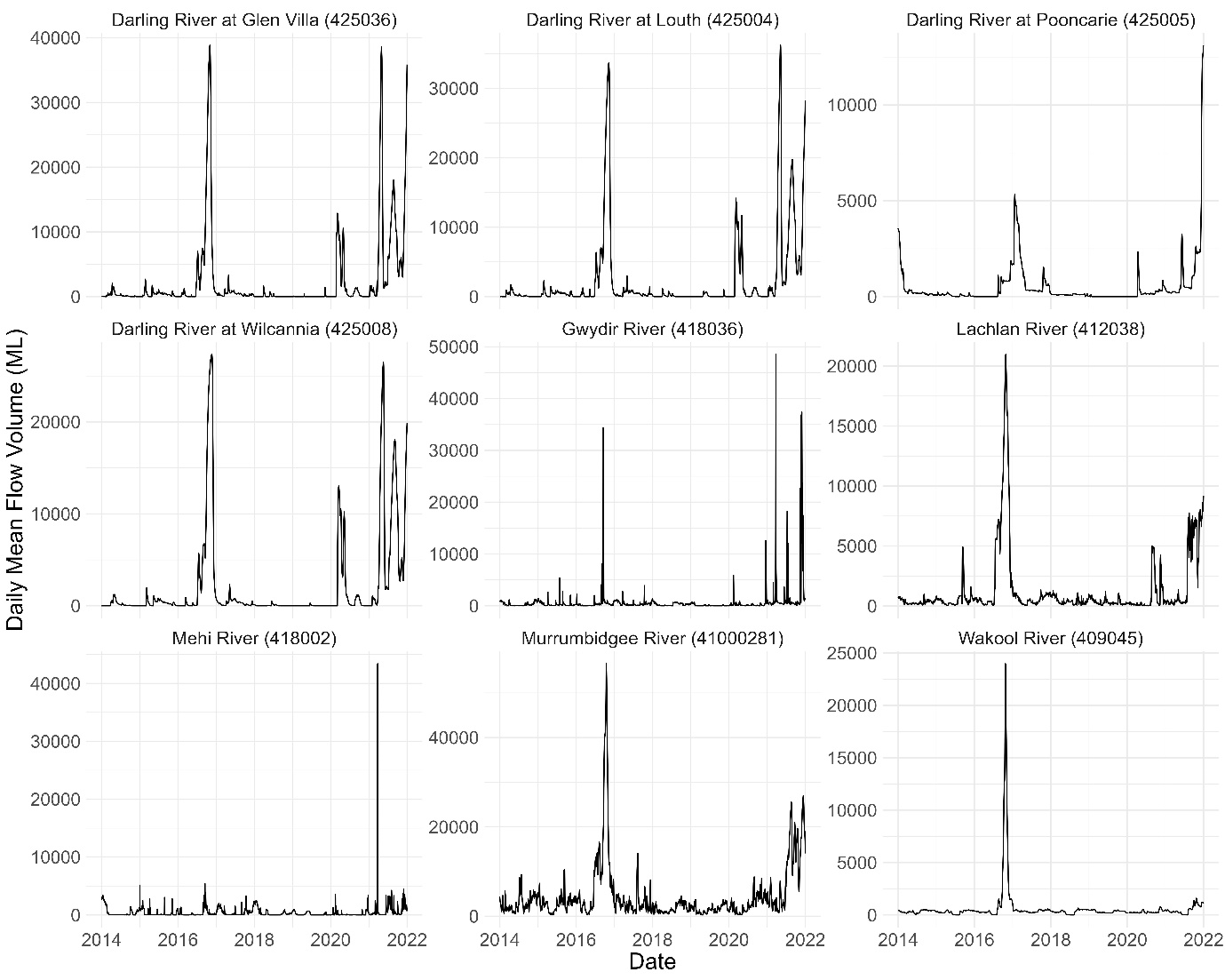
**

Supplementary Figure S1: Hydrographs for each flow gauge used to generate flow metrics for our valley-scale analysis. Flooding at multiple locations occurred in 2016, 2020 and 2021 and drought occurred in 2017 – 2019 (Bureau of Meteorology, 2020).

Supplementary Material S1: River descriptions and hydrology

The Gwydir and Mehi Rivers are located in northern NSW, generally flowing westwards with lengths of 480 km and 314 km respectively. The Mehi river is an anabranch of the Gwydir, and flows into the Barwon River, whilst the Gwydir terminates in a series of ephemeral wetlands that only connect with the Barwon during extreme flood events. Copeton Dam is the major reservoir within the system, and sits approximately 100 km south-east and upstream of where the Mehi and Gwydir rivers diverge. Below this, Tareelaroi Weir then controls diversions to the Mehi river. The Gwydir River system is considered a relatively degraded river and in poor ecological health (Davies et al., 2010; Hladyz et al., 2022).

The Darling River officially starts in northern NSW at the confluence of the Barwon and Culgoa Rivers. It runs in a south-westerly direction until it reaches the Menindee Lake system, a series of shallow natural lakes, from where it is referred to as the lower Darling catchment. The upper section of the Darling River (above Menindee Lakes) has no major water storages, but has numerous small and medium-sized weirs. Below this, Menindee Lakes have been modified into water storages with the addition of several weirs, levees and channels.

The Lachlan River flows from east to west in the southern MDB (central NSW), terminating at the Great Cumbung Swamp. The river extends 1339 km and has an annual stream flow of 834 GL. During times of high flow, the Lachlan river forms a connection with the Murrumbidgee river. There are two main dams on the Lachlan River: Wyangla dam and Jemalong weir. The Lachlan river is generally considered to be in very poor ecological health, with most fish species severely impacted (Davies et al., 2010).

The Murrumbidgee River flows from east to west in the southern MDB (southern NSW and ACT), terminating at the Murray River. The Murrumbidgee is 1485 km in length and has an annual stream flow of 4000 GL. The Murrumbidgee is primarily regulated by Burrinjuck, Blowering, Talbingo, Tantangara and Googong dams. The Murrumbidgee is generally considered to be in poor ecological health with severely impacted species abundance and diversity (Davies et al., 2010).

The Wakool River is part of a complex system of rivers known as the Edward/Kolety-Wakool system in southern NSW and is an anabranch of the Edward River, with the Edward River being an anabranch of the Murray River. The Wakool River generally flows in a westward direction until connecting back to the Murray River (via the Edward River) after a course of 363 km. The system is generally considered to be in poor ecological health (Davies et al., 2010).

Supplementary Table S1: Gauge locations and numbers used for each river valley.

| **River** | **Flow Gauge** | **Weather station** |
| --- | --- | --- |
| Murrumbidgee River | Carrathool – 41000281 | Narrandera – 74148 |
| Lachlan River | Willandra – 412038 | Hillston – 75032 |
| Wakool River | Barham-Moulamien – 409045 | Deniliquin Airport – 74258 |
| Gwydir/Mehi River | Boolooroo – 418036  Moree – 418002 | Moree Airport - 053115 |
| Darling River | Glen Villa – 425036  Louth – 425004  Wilcannia – 425008  Pooncarie – 425005 | Bourke Airport – 048245  Wilcannia – 46043  Pooncarie – 047029 |

Supplementary Table S2: Tukey pairwise comparisons of predicted length quartiles for basin and valley-scale analyses.

|  | Bony herring | | Common carp | | Golden perch | | Murray cod | |
| --- | --- | --- | --- | --- | --- | --- | --- | --- |
| Quartiles | Estimate | p | Estimate | p | Estimate | p | Estimate | p |
| Basin-scale |  |  |  |  |  |  |  |  |
| Q1 - Q2 | -0.189 | **<0.05** | -0.097 | **<0.05** | -0.267 | **<0.05** | 0.241 | **<0.05** |
| Q1 - Q3 | -0.192 | **<0.05** | -0.112 | **<0.05** | -0.447 | **<0.05** | 0.156 | **<0.05** |
| Q1 - Q4 | -0.227 | **<0.05** | -0.059 | **<0.05** | -0.819 | **<0.05** | -0.073 | **<0.05** |
| Q2 - Q3 | -0.003 | >0.05 | -0.015 | >0.05 | -0.180 | >0.05 | -0.085 | **<0.05** |
| Q2 - Q4 | -0.038 | >0.05 | 0.038 | >0.05 | -0.552 | **<0.05** | -0.314 | **<0.05** |
| Q3 - Q4 | -0.035 | >0.05 | 0.053 | **<0.05** | -0.372 | **<0.05** | -0.229 | **<0.05** |
| Valley-scale |  |  |  |  |  |  |  |  |
| Q1 - Q2 | 0.059 | **<0.05** | -0.029 | >0.05 | -0.381 | **<0.05** | 0.269 | **<0.05** |
| Q1 - Q3 | -0.004 | >0.05 | 0.009 | >0.05 | -0.366 | **<0.05** | 0.170 | **<0.05** |
| Q1 - Q4 | -0.153 | **<0.05** | 0.141 | **<0.05** | -0.632 | **<0.05** | -0.258 | **<0.05** |
| Q2 - Q3 | -0.063 | **<0.05** | 0.038 | >0.05 | 0.016 | >0.05 | -0.099 | **<0.05** |
| Q2 - Q4 | -0.212 | **<0.05** | 0.170 | **<0.05** | -0.251 | **<0.05** | -0.527 | **<0.05** |
| Q3 - Q4 | -0.149 | **<0.05** | 0.132 | **<0.05** | -0.266 | **<0.05** | -0.427 | **<0.05** |

# References

Bureau of Meteorology. (2020). *Special Climate Statement 70 update: drought conditions in Australia and impact on water resources in the Murray-Darling Basin*.

Davies, P., Harris, J., Hillman, T., & Walker, K. (2010). The sustainable rivers audit: assessing river ecosystem health in the Murray–Darling Basin, Australia. *Marine and Freshwater Research*, *61*(7), 764-777.

Hladyz, S., Baumgartner, L., Bice, C., Butler, G., Fanson, B., Giatas, G., Koster, W., Lyon, J., Stuart, I., Thiem, J., Tonkin, Z., Ye, Q., Yen, J., & Zampatti, B. (2022). *Basin-scale evaluation of 2020-21 Commonwealth environmental water: Fish*.
